# Binding Curve Viewer: Visualizing the Equilibrium and Kinetics of Protein–Ligand Binding and Competitive Binding

**DOI:** 10.1021/acs.jcim.4c00130

**Published:** 2024-05-08

**Authors:** Yu Du

**Affiliations:** † Department of Clinical Laboratory, The Second Affiliated Hospital of Jiaxing University, Huancheng North Road 1518, Jiaxing, Zhejiang 314000, China; ‡ The Key Laboratory, The Second Affiliated Hospital of Jiaxing University, Huancheng North Road 1518, Jiaxing, Zhejiang 314000, China

## Abstract

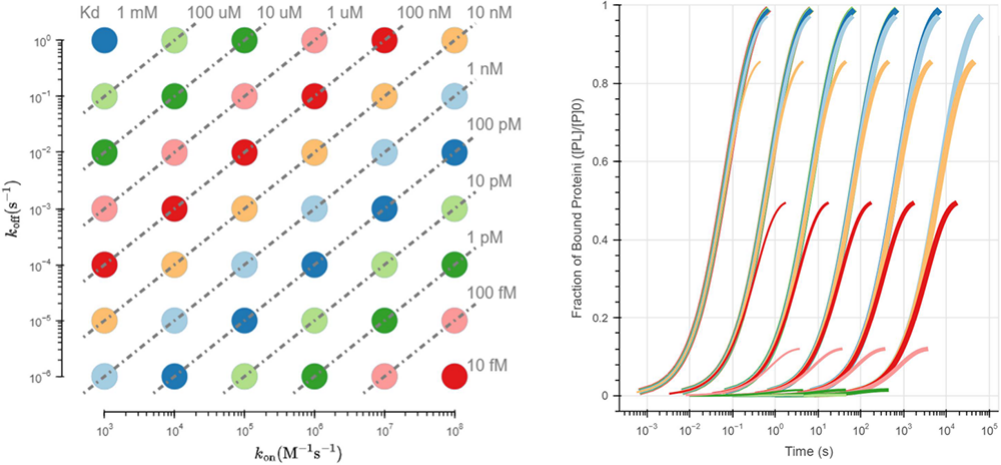

Understanding the thermodynamics and kinetics of the protein–ligand interaction is essential for biologists and pharmacologists. To visualize the equilibrium and kinetics of the binding reaction with 1:1 stoichiometry and no cooperativity, we obtained the exact relationship of the concentration of the protein–ligand complex and the time in the second-order binding process and numerically simulated the process of competitive binding. First, two common concerns in measuring protein–ligand interactions were focused on how to avoid the titration regime and how to establish the appropriate incubation time. Then, we gave examples of how the commonly used experimental conditions of [L]_0_ ≫ [P]_0_ and [I]_0_ ≫ [P]_0_ affected the estimation of the kinetic and thermodynamic properties. Theoretical inhibition curves were calculated, and the apparent IC_50_ and IC_50_ were estimated accordingly under predefined conditions. Using the estimated apparent IC_50_, we compared the apparent *K*_i_ and *K*_i_ calculated by using the Cheng–Prusoff equation, Lin–Riggs equation, and Wang’s group equation. We also applied our tools to simulate high-throughput screening and compare the results of real experiments. The visualization tool for simulating the saturation experiment, kinetic experiments of binding and competitive binding, and inhibition curve, “Binding Curve Viewer,” is available at www.eplatton.net/binding-curve-viewer.

## Introduction

Protein–ligand interactions in biological systems involve protein–protein, protein–nucleic acid, and protein–small molecule interactions. It is the protein–ligand interaction that underlies the biological phenomena and drug responses, such as the production or release of second messengers, cell proliferation, and growth inhibition of tumor cells. These interactions can be measured by various technologies, e.g., radioactive binding assays, fluorescence resonance energy transfer (FRET), isothermal titration calorimetry (ITC), and surface plasmon resonance (SPR).^1^ Quantitative properties derived from these assays include binding stoichiometry, dissociation constant (*K*_d_), association rate constant (*k*_on_), and dissociation rate constant (*k*_off_). These thermodynamic and kinetic parameters are essential for understanding the interaction between the protein and ligand. They provide insights into how many binding sites there are on the protein, how tightly the ligand binds to the protein, and how fast the binding or unbinding occurs. The binding assays, with labeled or unlabeled ligands, have greatly promoted the development of modern pharmacology, especially in the ligand identification of proteins and the screening and optimization of biomolecules.

In the protein–ligand interaction with a 1:1 stoichiometry and no cooperativity, the kinetics and thermodynamics of the binding species are of immense importance. There are three general options for assessing the affinity of the interaction: the equilibrium (or saturation) experiment, the kinetic experiment, and the competition experiment.^2^ In the saturation experiment and competition experiment, reaching the equilibrium of the interaction at the time of measurement is critical. The kinetic experiment, which is more revealing than the other two, can give both the rate constants and the equilibrium constant. After measuring the affinity of the labeled ligand for a target protein, competition experiments can be used to measure the affinities of other unlabeled ligands.^3^ Nowadays, a large amount of binding data have been reported in literature and deposited in data sets and databases, such as kinetic data of biomolecular interactions (KDBI),^4^ BindingDB,^5^ structural database of kinetics and energetics of mutant protein interactions (SKEMPI),^6^ kinetic and thermodynamic database of mutant protein interactions (dbMPIKT),^7^ kinetics of featured interactions (KOFFI),^8^ and PDBbind-koff-2020.^9^ Besides these databases of kinetic properties, there are even more databases of measured binding affinities, such as BindingDB,^5^ PubChem BioAssay,^10^ and ChEMBL,^11^ to name just a few.

As the binding data accumulates in public databases, problems like incorrect chemical structures,^12^ unit-transcription errors,^13^ and data inaccuracy,^14^ have been pointed out and partially solved.^15^ Robust biomolecular screening and structure–activity relationship (SAR) studies rely on the high-confidence binding data generated by experimental scientists. Therefore, from the perspective of experimentation, several publications have emphasized the importance of well-designed assays in obtaining accurate binding data.^2,16−18^ These publications focus on experimental strategies and factors affecting data interpretation in binding assays: the binding experiment must approach equilibrium at the time of measurement; in the equilibrium experiment, if the fixed and low concentration of protein or ligand is higher than the dissociation constant, then the experiment will be affected by titration. In this assay, we shall visualize the saturation experiment and the dynamics of protein–ligand binding and competitive binding by simulation to assist researchers in experiment planning and validation.

The simulation, visualization, and fitting of binding and reaction curves have been studied by numerous researchers. Their studies have advanced our understanding of the binding processes and enzymatic reactions. For example, Shave et al. implemented PyBindingCurve, a Python package, to simulate, plot, and fit the formation and inhibition of homodimers and heterodimers. Their code can be used to parse custom-defined systems of 1:*n* binding (*n* is 1–5) and 1:1:1 competition and solve the systems as constrained optimization problems. By the visualization of numerical simulation, the authors observed different behaviors between homodimer and heterodimer in the dimerization and dimer breaking in the presence of an inhibitor.^19^ More recently, Pääkkönen et al. presented a set of web-based simulation applets that can visualize the binding curves of equilibrium under custom-defined conditions in the experiments of dimerization, receptor–ligand binding, and competitive binding. Users can set the concentrations of binding species and the dissociation constants of different complexes to calculate the species abundances at equilibrium. The authors also provided a curve-fitting tool to estimate the unknown dissociation constants using users’ input data.^20^ In the extensive history of simulating and fitting the enzyme kinetics, numerous tools and software packages have been developed, such as KINSIM,^21^ KinTek,^22^ DynaFit,^23^ ENZO,^24^ and ICEKAT,^25^ to name a few. These tools mainly focus on the selection of complex enzymatic models and fitting rates of enzyme reactions, which has been summarized in the excellent review.^26^ In the field of biochemical education, dynamic visualization is also preferred in the classroom,^27−29^ which can enhance students’ knowledge of the enzyme kinetics and binding interaction. Although the above-mentioned tools can be used to simulate the equilibrium concentrations of analytes at varied concentrations of the protein and ligand, to our knowledge, there are no available tools directly showing the difference between *K*_d_ and apparent *K*_d_, the differences between second-order and pseudo-first-order kinetics, the kinetics of the competitive binding, and the theoretical inhibition curve (see Table 1 for computational tools used in binding assay). These functions may significantly facilitate the planning and validation of binding experiments.

**Table 1:** Implementation and Functionality of Different Computational Tools Used in the Binding Assay

tools	implementation	notes
Binding Curve Viewer	Web server; Program	visualizes the equilibrium and kinetics of protein–ligand binding and competitive binding
PyBindingCurve^19^	Program	simulation and curve fitting to complex binding systems at equilibrium
CLAffinity^30^	Program	identifies optimum ligand affinity for competition-based primary screens
protsim^20^	Web server	visualizes equilibrium reactions of protein homodimerization and competitive ligand binding
pharmechanics^31^	Microsoft Excel; GraphPad Prism	provides Excel add-in and Prism protocol for pharmacology data analysis
equilibrium expert^32^	Microsoft Excel	simulates multiple binding equilibrium
IC_50_-to-*K*_i_^33^	Web server	calculates *K*_i_ from IC_50_ using Michaelis–Menten kinetic equation or nonclassic equation for different inhibitory mechanisms
*K*_i_ calculator^34^	Microsoft Excel	calculates *K*_i_ from IC_50_ using Wang’s group equation

To visualize the equilibrium and kinetics of the binding reaction with 1:1 stoichiometry and no cooperativity, we solved the differential equations of the concentration of the protein–ligand complex and the time in the second-order binding process and numerically simulated the process of the competitive binding. The theoretical inhibition curves were calculated and the apparent IC_50_ and IC_50_ were estimated accordingly under predefined conditions. Based on these works, the “Binding Curve Viewer” is presented herein as a set of web-based interactive visualizations for binding curves of the equilibrium experiment, kinetic experiment, and competition experiment. The graphs in the Binding Curve Viewer can be updated in real time when users alter the parameters of the thermodynamics, kinetics, and concentrations of the protein, ligand, and inhibitor. Binding Curve Viewer can show the difference between *K*_d_ and apparent *K*_d_ to help researchers avoid the titration regime. The analytical and numerical integration of the differential equations of binding and competitive binding gave accurate time to reach 99% equilibrium for researchers choosing a proper incubation time. The simulated graphs, akin to experimental binding curves, may shed light on the affinity and dynamics of the protein–ligand interaction for experimental planning and result validation. A simulation tool for competition-based high throughput screening (HTS) is also demonstrated in our work. Binding Curve Viewer is an open-source webpage and software that is accessible over the Internet and can be downloaded for offline usage.

## Methods

### Overview of the Binding Curve Viewer

“Binding Curve Viewer” is an open-source web application and software for visualization of the equilibrium and kinetics of protein–ligand binding and competitive binding. Binding Curve Viewer (the webpage) is a set of interactive applications and available at https://eplatton.net/binding-curve-viewer under the CC-BY 4.0 license. The webpage depends on HTML, JavaScript, and BokehJS. The webpage supports modern browsers (e.g., Chrome/Chromium browser 94+ or equivalent) on a wide variety of UNIX platforms, Windows, and MacOS. The webpage is the output of Binding Curve Viewer (the code). The code is in the Python module *binding_curve_viewer.py* with multiple functions under the MIT License. The code depends on Python 3.7.6 or higher and Python packages (Bokeh 3.4.0 or higher and Numpy 1.26.0 or higher). The code can be downloaded at https://github.com/ydu-sci/Binding_Curve_Viewer. Users can generate the webpage according to the instructions in README.rst. To guide the usage of the code, we wrote detailed documentation in the code and made HTML files using Python documentation tools, Sphinx 5.0.2 or higher, and guzzle_sphinx_theme. Below, we describe the implementation of the main functions of the code. It is worth noting that, in the work, the binding between the protein and ligand or inhibitor was 1:1 stoichiometry without cooperativity. The experimental conditions of the temperature, pH, and ionic strength were not considered and were supposed to be constant.

### Determination of the Theoretical Saturation Curve and the Apparent *K*_d_

To determine the dissociation constant of the interaction between the protein and ligand, one component was in fixed and limited concentration and titrated by the other component until the fixed component was near-fully bound. In common practice, the fixed component interacts with a series of reasonable concentrations of the other component. Here, we specify that the protein was the component in a fixed concentration. The ligand can be any molecule, e.g., DNA, RNA, or peptides, that binds to the target protein. The dissociation constant, *K*_d_, is defined by eq 1,
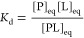
1where [P]_eq_ is the equilibrium concentration of the free protein, [L]_eq_ is the equilibrium concentration of the free ligand, and [PL]_eq_ is the equilibrium concentration of the protein–ligand complex. According to eq 1, when half of the total protein is bound to the ligand at equilibrium, and the other half of the total protein is free ([PL]_eq_ = [P]_eq_), the [L]_eq_ equals *K*_d_. At the same time, the concentration of the total ligand ([L]_total_) equals the apparent *K*_d_. According to the user-defined *K*_d_ and concentration of the total protein ([P]_total_), the following equation derived from eq 1 was used to generate the saturation curve by a step size sequence of [PL] from protein being unbound to near-fully bound, i.e., [0, [P]_total_).
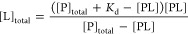
2The total concentration of the protein and the total concentration of the ligand were the working concentrations of each of them in the binding reaction initiated by mixing an arbitrary volume of protein with an arbitrary volume of ligand. By default, 1000 points were calculated for this curve, and the number of points determined the smoothness of the curve. To avoid the long tail near the end of the titration, only 970 points were shown.

### Kinetics of Association and Dissociation

In the second-order binding process of the protein and ligand, the binding kinetics describe the rate of association of the protein and ligand with each other and the dissociation of the protein–ligand complex. This process can be described by the following equation
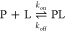
3At equilibrium, the association rate (*k*_on_ × [P]_eq_ × [L]_eq_) equals the dissociation rate (*k*_off_ × [PL]_eq_). With eq 1, the dissociation constant equals the ratio of the first-order dissociation rate constant (*k*_off_) to the second-order association rate constant (*k*_on_),
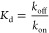
4Second-order kinetics is usually converted to pseudo-first-order kinetics by using the ligand in large excess over the protein. In this scenario, the concentration of the free ligand remains approximately constant (the same as the initial concentration of the ligand, [L]_0_) during the binding process; then, the binding process reduces to
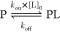
5To plot the concentration changes over time, we solved the differential equation to obtain the exact relationship between the concentration of the protein–ligand complex and the time in the second-order binding process (see the solution of the differential equation in Supporting Information, hereafter referred to as SI).
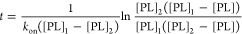
6where [PL]_1_ and [PL]_2_ are the roots of a quadratic equation and they can be calculated from [P]_0_, [L]_0_, and *K*_d_ ([P]_0_ and [L]_0_ is the initial concentration of the protein and ligand, see SI for the expressions of [PL]_1_ and [PL]_2_). In the pseudo-first-order binding process, the relationship between the concentration of the protein–ligand complex and the time is
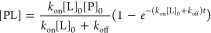
7The initial concentration means the working concentration in the binding reaction initiated by mixing an arbitrary volume of protein with an arbitrary volume of ligand. According to the user-defined [P]_0_, [L]_0_, *k*_off_, and *k*_on_, the association curve of the second-order binding process was generated by using eq 6 and a step size sequence of the [PL] from 0 to the equilibrium concentration ([PL]_eq_). In the second-order binding process, [PL]_eq_ can be calculated by the kinetic and thermodynamic approaches, both resultant equations were the same (see the derivation in SI),
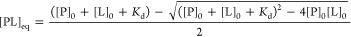
8The association curve of the pseudo-first-order binding process was generated by using eq 7 and the resultant sequence of the time from the second-order binding process. This operation aligned the time points of both curves to facilitate vertical hover tools. In the pseudo-first-order binding process, [PL]_eq_ calculated by the thermodynamic approach was the same as [PL]_eq_ calculated by the kinetic approach (see the derivation in SI),
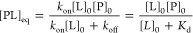
9

We plotted the dissociation curve under ideal conditions where no rebinding occurred. The common practices, dilution or adding an unlabeled compound, are used to avoid rebinding. In this dissociation process, the relationship of the [PL] and the time is (see the derivation in the SI),
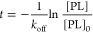
10

The dissociation curve was generated by using eq 10 and a step size sequence of [PL] from [PL]_eq_ to 0, i.e., (0, [PL]_eq_]. [PL]_0_ equaled [PL]_eq_ calculated from the second-order binding process under the user-defined condition. By default, 1000 points were calculated for the association and dissociation curve, the number of points determined the smoothness of the curve. To avoid the long tail near the end of the association and dissociation curves, only 990 points were shown.

### Estimation of the Kinetic Properties from the Association Kinetic Experiment

**Figure 1:**
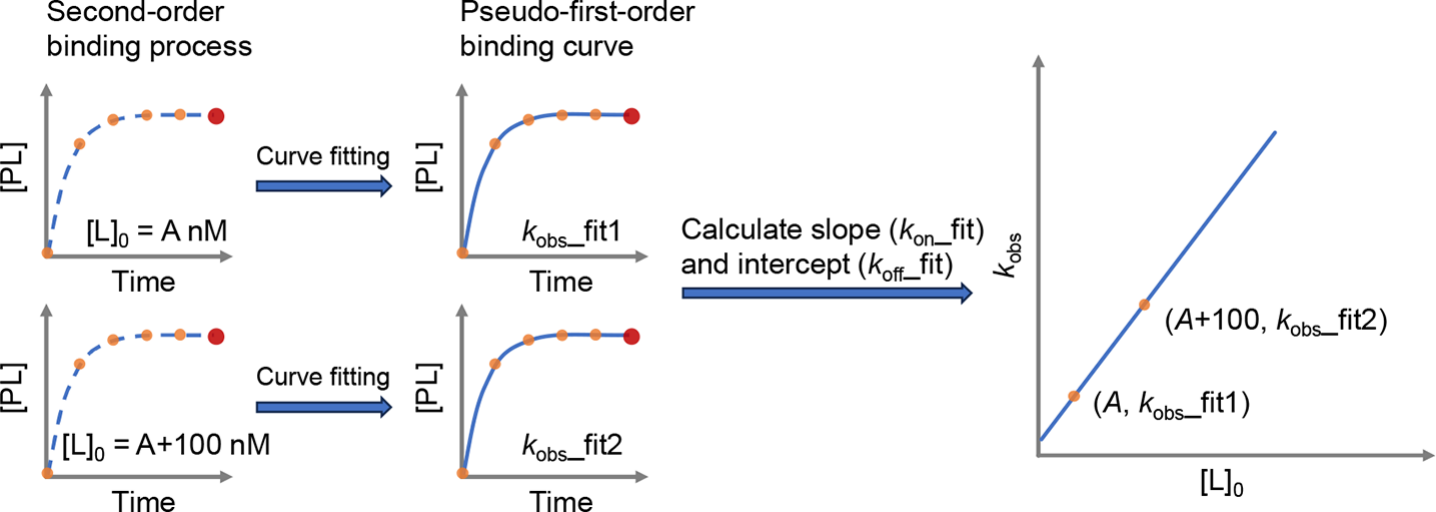
Schematic diagram of the process of predicting *k*_obs_, *k*_off_, and *k*_on_. In the conditions of [P]_0_ = 100 nM, *k*_off_ = 0.01 s^–1^, and *k*_on_ = 1e5M^–1^ s^–1^, five measurements and the zero point of the [PL] from the second-order binding process were fitted by the pseudo-first-order binding curve. End-point measurements are shown in red and slightly larger circles, others are shown in orange circles. *k*_obs__fit1 and *k*_obs__fit2 were calculated by two measurements with the difference between [L]_0_ being 100 nM. *k*_on__fit and *k*_off__fit can be obtained from the slope and intercept of the line formed by the coordinates of (*A*, *k*_obs__fit1) and (*A*+100, *k*_obs__fit2).

In the common practice of the association kinetic experiment, the ligand is in large excess over the protein. The measurements of the [PL] at different times in the second-order binding process can be fitted by the pseudo-first-order binding curve, i.e., estimating the parameter of *k*_obs_. The experimental conditions throughout the estimation of the kinetic properties were [P]_0_ = 100 nM, *k*_off_ = 0.01 s^–1^, *k*_on_ = 1e5 M^–1^ s^–1^, and different [L]_0_. As Figure 1 shows, six evenly separated measurements (including the zero point) were simulated by a second-order binding process. The end-point measurement was a certain percentage of the equilibrium, by default it was 99% of the equilibrium. These six points were fitted by the pseudo-first-order binding curve (eq 1.11 in the SI). A *k*_obs__fit would be calculated for each [L]_0_. As Figure 1 shows, for two different [L]_0_, a linear equation could be determined by two points whose coordinates were (*A*, *k*_obs__fit1) and (*A*+*N*, *k*_obs__fit2). By default, the difference in the ligand concentrations ([L]_0_) between the two measures was 100 nM (*N* = 100 nM). Then, *k*_on__fit and *k*_off__fit could be calculated by solving the slope and intercept of the linear equation. The data generation and fitting process was programmed in the Python script *association_curve_fitting.py* in the source code.

### Calculation Methods of the Competitive Binding of Association and Dissociation


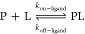
11
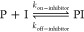
12

The competitive binding is described by eqs 11 and 12. The equilibrium state of the competitive binding of the ligand and inhibitor to one binding site of a protein molecule with no cooperativity can be exactly calculated by the Wang equation from the total concentrations of the protein, ligand, and inhibitor ([P]_0_, [L]_0_, and [I]_0_), the dissociation constant of the protein and ligand (*K*_d_), and the dissociation constant of the protein and inhibitor (*K*_i_).^35^ There is no usage restriction in the total concentrations of the protein, ligand, and inhibitor, e.g., [L]_0_ ≫ [P]_0_ or [I]_0_ ≫ [P]_0_. Therefore, we term the equilibrium concentration calculated by the Wang equation the “theoretical equilibrium concentration” in the second-order binding process. It should be emphasized that the consistent unit of concentration used in our program was nanomolar, we did not encounter the round-off errors in the Wang equation shown in the work of Blay et al.^36^

To describe the kinetic process of competitive binding (eqs 11 and 12), we used the Motulsky–Mahan equation^37^ to calculate the relationship of [PL] and time in the association process. By association, we mean that the binding is initiated by mixing an arbitrary volume of protein with an arbitrary volume of the mixture of ligand and inhibitor. The Motulsky–Mahan equation only applies to the limited experimental conditions: (1) the initial concentrations of the protein–ligand complex and protein-inhibitor complex ([PL]_0_ and [PI]_0_) should be 0; (2) “only a small fraction (<10%)”^37^ of the ligand and inhibitor bind to the protein; thus, [L]_eq_ and [I]_eq_ approximately equal [L]_0_ and [I]_0_, respectively. Hence, the Motulsky–Mahan equation can only be applied in the pseudo-first-order binding process.

The Motulsky–Mahan equation cannot describe the competitive binding of dissociation. By dissociation, we mean that the competitive binding is initiated by mixing the equilibrated protein and ligand with the inhibitor in a specific volume ratio. Thus, the equilibrated protein–ligand system will be disrupted by the volume change and competition of the inhibitor. We devised the following numerical method to simulate the second-order kinetic process of the competitive binding of both association and dissociation. The *n* in the equations is a positive integer,
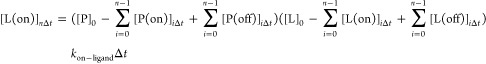

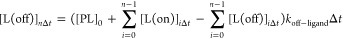

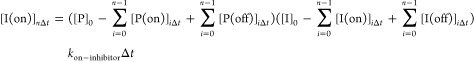

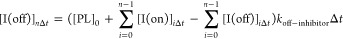

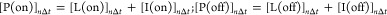

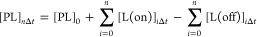


For the ligand and inhibitor, the competitive binding process of each association and dissociation can be explicitly divided into the “on” and “off” subprocesses. In the competitive binding of association, [P]_0_, [L]_0_, and [I]_0_ were the working concentrations after mixing, and [PL]_0_ and [PI]_0_ equaled 0. In the competitive binding of dissociation, [P]_0_, [L]_0_, [I]_0_, and [PL]_0_ were the instantaneous concentrations after mixing by some volume ratio, and [PI]_0_ equaled 0. [P(on/off)], [L(on/off)], and [I(on/off)] were defined as the concentrations of newly disappeared/generated protein, ligand, and inhibitor in the interval of time, Δ*t*. We assumed that if Δ*t* was small enough, during Δ*t*, the concentrations of the protein, ligand, and inhibitor were hardly affected by others’ on-and-off subprocess. In the competitive binding process of both association and dissociation, timing begins at the moment of mixing.

To avoid the possible stiffness in the simulation,^38^ we divided the simulation into two consecutive phases. First, we calculated the time to reach 99% equilibrium (*t*_0.99-ligand_) of the association of only protein and ligand in the competitive binding of association or the time to reach 99% equilibrium (*t*_0.99-ligand_) of the dissociation of the only protein–ligand complex in the competitive binding of dissociation. The *t*_0.99-inhibitor_ value of the association of only the protein and inhibitor was also calculated. Then, the smaller one of *t*_0.99-ligand_ and *t*_0.99-inhibitor_ was used as the duration of the first phase, e.g., *t*_0.99-ligand_, 10 times the larger one was used as the end of the second phase, from *t*_0.99-ligand_ to 10 × *t*_0.99-inhibitor_. In each phase, Δ*t* equaled the duration divided by 200,000, *t*_0.99-ligand_/200,000 in the first phase and (10 × *t*_0.99-inhibitor_ – *t*_0.99-ligand_)/200,000 in the second phase. The denominator 200,000 determined the discretization error in the numerical simulation. This parameter cannot be changed in the webpage, but users can increase this parameter in the program and generate a new webpage to obtain a finer simulation. By default, we used 200,000 and 400,000 in the competitive binding of association and dissociation, respectively, for the reliable approximation of the kinetic processes in a reasonably short time of calculation (see Tables S1 and S2 for the error analysis of the simulation). It is worth noting that, in the competitive binding experiment, *t*_0.99_ was calculated by the time point closest to the 99% equilibrium in the second-order and pseudo-first-order process. At the end of the two phases, the concentrations of each species were defined as the equilibrium concentrations in the simulation.

### Calculation Methods of the Theoretical Inhibition Curve, Apparent IC_50_, and IC_50_

Based on Wang’s work,^35^ the theoretical inhibition curve can be calculated for any inhibitor by using specific concentrations of the total protein and ligand ([P]_0_ and [L]_0_), *K*_d_, and *K*_i_, if *K*_i_ of the protein and inhibitor is predefined. First, we estimated IC_50_ to determine the range of the total concentration of the inhibitor ([I]_0_). We used the Cheng–Prusoff equation^3^ to estimate IC_50_ from the known [L]_0_, *K*_d_, and *K*_i_, e.g., the estimated IC_50_ = A. Then, we gradually increased [I]_0_ in the range of [0, 100 × A] and used the Wang equation^35^ to calculate the equilibrium concentration of the protein–ligand complex ([PL]_eq_) and the equilibrium concentration of the free inhibitor ([I]_eq_) under the condition of each [I]_0_. When the calculation was completed in the full range of [0, 100 × A], IC_50_ and apparent IC_50_ can, respectively, be approximated by [I]_eq_ and [I]_0_, at each concentration was closest to 50% inhibition of the “initial binding” of protein and ligand (see Zhang et al.’s work^39^ for the caveats when using these parameters). Hence, the resolution of the range will determine the accuracy of the estimated IC_50_ and apparent IC_50_. The initial binding means the equilibrium concentration of the protein–ligand complex in the blank control in the competitive binding of either association or dissociation. In association, the blank control was the mixture of the protein and the solution with ligand and no inhibitor. In dissociation, the blank control was the mixture of the equilibrated protein and ligand and the solution without inhibitor. The blank control is important to eliminate the equilibrium concentration change of the protein–ligand complex upon the volume change after mixing. Wang’s group derived an exact mathematical relationship of the apparent IC_50_ and *K*_i_ for most competitive binding experiments.^34^ Using the Cheng–Prusoff equation,^3^ the Lin–Riggs equation,^40^ and Wang’s group equation,^34^ we calculated the apparent *K*_i_ or *K*_i_ from the apparent IC_50_ estimated from the theoretical inhibition curve (see Section 8 in SI for details).

## Results and Discussion

### *K*_d_ and Apparent *K*_d_ Can be Shown in Theoretical Saturation Curves

**Figure 2:**
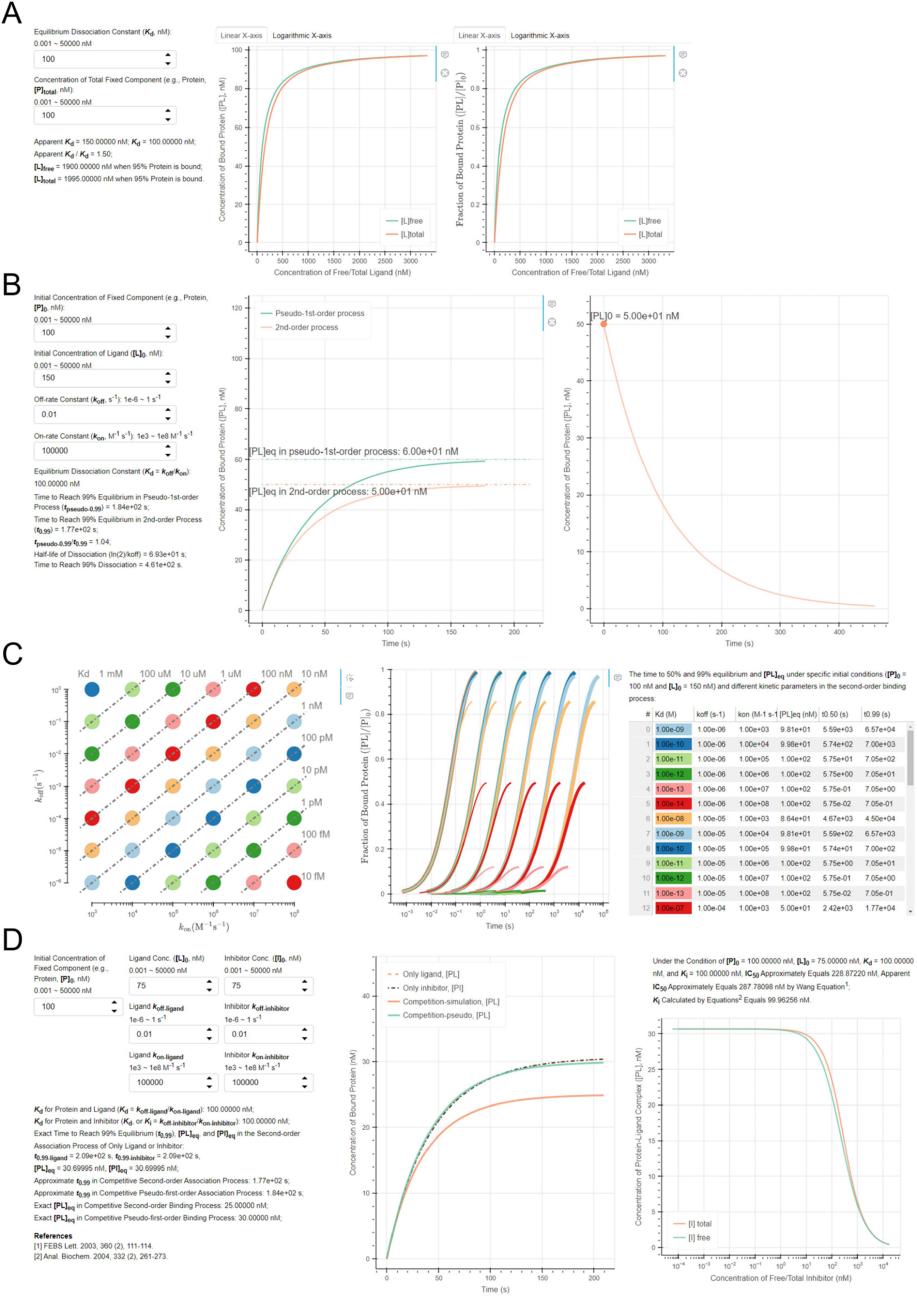
Screenshot of the functionality of Binding Curve Viewer. (A) Determination of the dissociation constant. (B) Kinetics of association and dissociation. (C) Iso-affinity graph. (D) Competitive binding kinetics association. We omitted the functionality, the competitive binding kinetics -dissociation, due to the limited image size, see the webpages for all the details.

Users can change the value of the equilibrium dissociation constant, *K*_d_, and the concentration of the total fixed component (e.g., the protein, [P]_total_) (Figure 2A). Both values can be input ranging from 0.001 to 50,000 nM. By default, *K*_d_ equaled 100 nM, and [P]_total_ equaled 100 nM. Under this condition, the theoretical saturation curve was calculated. The *x*-axis was the concentration of the free or total ligand on either a linear or logarithmic scale. The apparent *K*_d_ was calculated to be 150 nM and the ratio of the apparent *K*_d_ to *K*_d_ was 1.50. When 95% of the total protein was bound, [L]_total_ was 1995.0 nM, this value can be used to design the concentration series in titration experiments if researchers have known the approximate *K*_d_ from preliminary experiments.

The divergence between *K*_d_ and apparent *K*_d_ was more obvious when the concentration of the constant limited component, e.g., the protein in our case, was higher than *K*_d_. The concentration of the bound or free ligand can be measured by different experimental protocols. If the concentration of the bound ligand is measured, researchers usually assume that the resultant apparent *K*_d_ can be used to properly approximate *K*_d_. To check for the titration regime, users can input [P]_total_ and *K*_d_ to compare the apparent *K*_d_ with *K*_d_ after measurements. To estimate the *K*_d_ by the apparent *K*_d_, we recommend that the ratio of the apparent *K*_d_ to *K*_d_ should be less than 1.05 under the proper concentration of the limited component, whose lower limit of detection, on the other hand, may have been set by the experimental protocols. Therefore, by using the “Binding Curve Viewer”, we show how and to what extent the concentration of the fixed component affects the apparent *K*_d_.

### Kinetics of the Association and Dissociation of the Protein and Ligand

Users can change the initial concentration of the protein ([P]_0_), the initial concentration of the ligand ([L]_0_), the dissociation rate constant (*k*_off_), and the association rate constant (*k*_on_). [P]_0_ and [L]_0_ can be input ranging from 0.001 to 50,000 nM. The range of *k*_off_ and *k*_on_ is, respectively, 1e-6∼1 s^–1^ and 1e3∼1e8 M^–1^ s^–1^. The association and dissociation curves are shown alongside the parameter panel (Figure 2B). With this tool, ligands with different kinetic properties can be compared (see Table S3 for more information). The hypothetical experiments in Table S3 show that the binding process can be obviously accelerated by using a large [L]_0_ or a ligand with a large *k*_on_.

**Figure 3:**
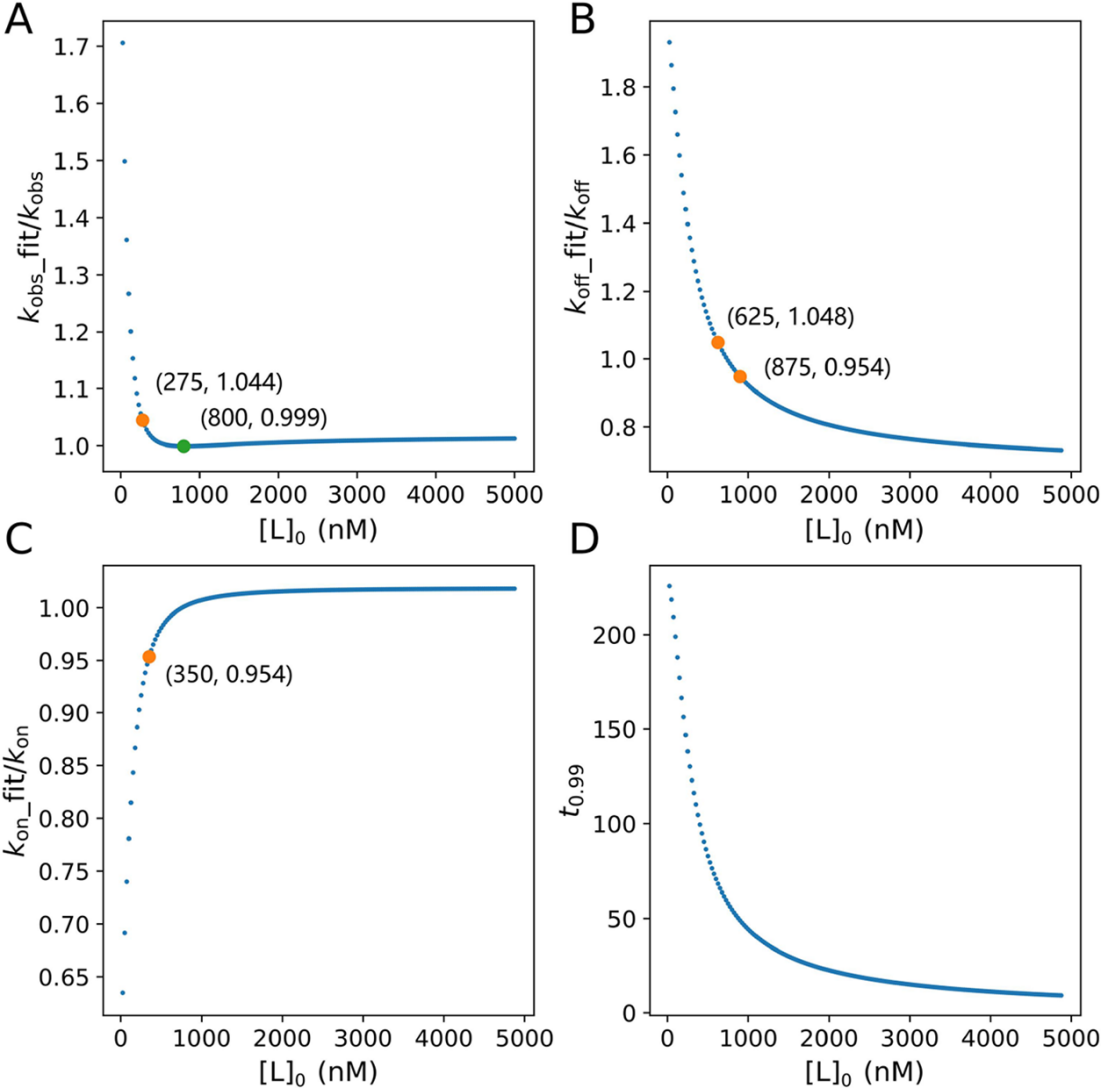
Relationship of [L]_0_ and the ratio of the predicted (A) *k*_obs_, (B) *k*_off_, and (C) *k*_on_ to their theoretical values. The orange point indicated the end points of each ratio in the range 1.000 ± 0.050. The green point indicated the minimum point of *k*_obs__fit/*k*_obs_. The corresponding coordinates were displayed near these points. (D) Relationship of [L]_0_ and the time to 99% equilibrium of the association process (*t*_0.99_).

To demonstrate how the changed [L]_0_ affected the estimation of *k*_obs_, *k*_off_, and *k*_on_ and the proper range of [L]_0_ for the estimation, we analytically simulated the association kinetic experiments in the condition of [P]_0_ = 100 nM, *k*_off_ = 0.01 s^–1^, *k*_on_ = 1e5 M^–1^ s^–1^, and different [L]_0_ (Figure 3). The end-point measurement was 99% of the equilibrium. It is worth noting that we used the exact values of [PL] at different times in the second-order binding process without introducing random errors during the simulation. *k*_obs_ was estimated by nonlinear regression of the pseudo-first-order binding curve fitting. As [L]_0_ increased from 25 to 5000 nM by the step of 25 nM, the ratio of *k*_obs__fit to *k*_obs_ first steeply declined toward 1. When [L]_0_ equaled 275 nM, *k*_obs__fit/*k*_obs_ equaled 1.044, which was below 1.050 for the first time. When [L]_0_ increased to 800 nM, *k*_obs__fit/*k*_obs_ decreased to the minimum value of 0.999, then gradually increased to 1.013 when [L]_0_ increased to 5000 nM (Figure 3A). We calculated the slope (*k*_on__fit) and intercept (*k*_off__fit) of the line formed by (*A*, *k*_obs__fit1) and (*A*+100, *k*_obs__fit2), *A* was in the range of [25, 4900] with a step size of 25 nM. As [L]_0_ increased from 25 to 4900 nM, *k*_off__fit/*k*_off_ decreased from 1.931 to 0.729 (Figure 3B). When [L]_0_ was in the range of 625 to 875 nM, *k*_off_ could be accurately predicted, as *k*_off__fit/*k*_off_ was in the range of 1.000 ± 0.050. Figure 3C shows that as [L]_0_ increased from 25 to 4900 nM, *k*_on__fit/*k*_on_ increased from 0.634 to 1.018. When [L]_0_ was greater than 350 nM, *k*_on__fit/*k*_on_ was in the accurately predicted range (1.000 ± 0.050). Therefore, the above results show that the narrowest range [625, 875] of [L]_0_ may be the most proper concentration for accurately estimating *k*_obs_, *k*_off_, and *k*_on_ simultaneously in our constrained experimental condition. If [L]_0_ is greater than 875 nM, *k*_off_ may be underestimated. If *k*_off_ has been separately determined by the dissociation kinetic experiments, then the estimation of *k*_on_ will not be affected because *k*_on__fit is determined by *k*_obs__fit and [L]_0_, not the value of *k*_off_.

Additionally, researchers also need to consider the time to 99% equilibrium of the association process (Figure 3D). Figures S1–S3 demonstrate that the end-point measurements can significantly affect the estimation of *k*_obs_, *k*_off_, and *k*_on_. If the end-point measurements of [PL] were below 96% of the equilibrium (96% × [PL]_eq_) in the kinetic experiment, *k*_obs_ could not be confidently estimated. The confident ranges of [L]_0_ for estimating *k*_off_ and *k*_on_ did not overlap each other, either. This pre-equilibrium termination of the kinetic experiment led to an overestimation of *k*_obs_ and *k*_on_ (see the ratios of estimated values to theoretical values in Figures S1 and S3). Also, we increased the spacing between the two concentrations of [L]_0_ in the calculation of *k*_off__fit and *k*_on__fit. This spacing had no obvious effects on the estimation (Figures S4 and S5). In practice, the spacing needs to be properly large enough for the measurements to be distinct from the random errors under a serial of [L]_0_.

When plotting the dissociation curve, we assumed that there was no rebinding in the dissociation process (Figure 2B). The initial concentration of the protein–ligand complex ([PL]_0_ = 50 nM) was the equilibrium concentration in the second-order binding process.

### Examples of Association Kinetics in the Iso-Affinity Graph

To further compare different kinetic properties under the conditions of [P]_0_ = 100 nM and [L]_0_ = 150 nM, we generated the iso-affinity graph of the second-order binding process (Figure 2C). This tool had three parts: (1) The left part of Figure 2C. The *y*-axis and *x*-axis were *k*_off_ and *k*_on_ on a logarithmic scale, and circles on each diagonal represented ligands with the same *K*_d_. Circles on the left part of the diagonal represented slow-on–slow-off ligands and circles on the right part of the diagonal represented fast-on–fast-off ligands. In the iso-affinity graph, *k*_on_ could equal 1e3, 1e4, 1e5, 1e6, 1e7, or 1e8 M^–1^ s^–1^, and *k*_off_ could equal 1, 1e-1, 1e-2, 1e-3, 1e-4, 1e-5, or 1e-6 s^–1^; thus, *K*_d_ could range from 10 fM to 1 mM; (2) The middle part of Figure 2C. Each association curve was shown from the start of the binding to 99% equilibrium. The curves on the right-hand side had a longer time to reach 99% equilibrium; (3) The right part of Figure 2C. A table showed all cases with detailed information, e.g., *K*_d_, *k*_off_, *k*_on_ [PL]_eq_, and the time to 50 and 99% equilibrium. The curves and table can be updated upon users’ selection on the iso-affinity graph by clicking their interested circles. By default, all circles were selected, all curves, and all data in the table were shown.

With this tool, we could compare the binding processes of a constant *k*_on_ and changing *k*_off_. The ligands with smaller *k*_off_ values showed a longer time to reach 99% equilibrium (Figure S6). These phenomena may be explained by a longer time for a greater number of binding events in the stronger-affinity interaction. If we focused on the cases on the diagonal with the same *K*_d_, slow-on–slow-off ligands needed a longer time to reach 99% equilibrium (Figure S7). If we kept *k*_off_ constant, the larger the *k*_on_ was, the less time was required to reach 99% equilibrium (Figure S8).

### Competitive Binding Processes of Association and Dissociation Were Analyzed by Analytical and Numerical Integration

In the parameter panel of the webpage of the competitive binding (Figure 2D), users can change the initial concentration of the protein, ligand, and inhibitor ([P]_0_, [L]_0_, and [I]_0_), the dissociation rate constant (*k*_off-ligand_ or *k*_off-inhibitor_) and association rate constant (*k*_on-ligand_ or *k*_on-inhibitor_) of the ligand and inhibitor, and the volume ratio of the protein and ligand to the inhibitor. [P]_0_, [L]_0_, and [I]_0_ can be input ranging from 0.001 to 50,000 nM. The range of *k*_off_ and *k*_on_ is respectively 1e-6–1 s^–1^ and 1e3–1e8 M^–1^ s^–1^. The volume ratio can be a floating number ranging from 1.0 to 1,000.0. The competitive binding process can be updated on users’ input values in real time.

The simulation of the competitive binding of association was initiated by mixing an arbitrary volume of protein with an arbitrary volume of the mixture of the ligand and inhibitor. The working concentration of each component at the time of mixing can be determined by users’ input values of [P]_0_, [L]_0_, and [I]_0_. In the simulation of the competitive binding of dissociation, [P]_0_, [L]_0_, and [I]_0_ were the concentrations before mixing. The simulation was initiated by mixing the equilibrated protein and ligand with the inhibitor in a certain volume ratio. First, the binding of the protein and ligand reached equilibrium, and the equilibrium concentration of the protein, ligand, and protein–ligand complex was, respectively, [P]_eq_, [L]_eq_, and [PL]_eq_. For example, the volume ratio of the protein and ligand to the inhibitor equaled 2. At the time of mixing, the instantaneous concentration of the protein, ligand, protein–ligand complex, and inhibitor was respectively [P]_eq_ × 2/3, [L]_eq_ × 2/3, [PL]_eq_ × 2/3, and [I]_0_/3. The system with these instantaneous concentrations was the starting point of the simulation of the competitive binding of dissociation. The calculation of new equilibrium concentrations after mixing was based on the total concentrations of protein, ligand, and inhibitor by using the Wang equation.^35^

We showed four time–concentration curves in the plot of the competitive binding of association for easier comparison of the concentrations of the bound protein or the protein–ligand complex in the presence of only the ligand, only the inhibitor, and both the ligand and inhibitor. The association process was calculated by the analytical integration (Competition-pseudo in Figure 2D) and numerical simulation (Competition-simulation in Figure 2D). The dissociation process was only calculated by the numerical simulation (Competition-simulation in the webpage “Competitive Binding Kinetics-Dissociation”). With these tools, the ligands and inhibitors with different kinetic properties can be compared in the competitive binding of both association and dissociation processes (see Table S4 for more information).

Under users’ input conditions, if the difference between the simulated [PL]_eq_ and theoretical [PL]_eq_ is greater than 0.1% of the theoretical [PL]_eq_, there will be a notice on the webpage, and users may increase the resolution and the duration of the simulation in the program and generate a new simulation themselves. In the simulation of the competitive binding of dissociation, if the concentration of free protein was relatively large at the time of mixing, the concentration of the protein–ligand complex would decrease slowly (see Section 7 in SI for details).

### Comparison of the Apparent *K*_i_ and *K*_i_ Calculated by Using Different Equations

**Figure 4:**
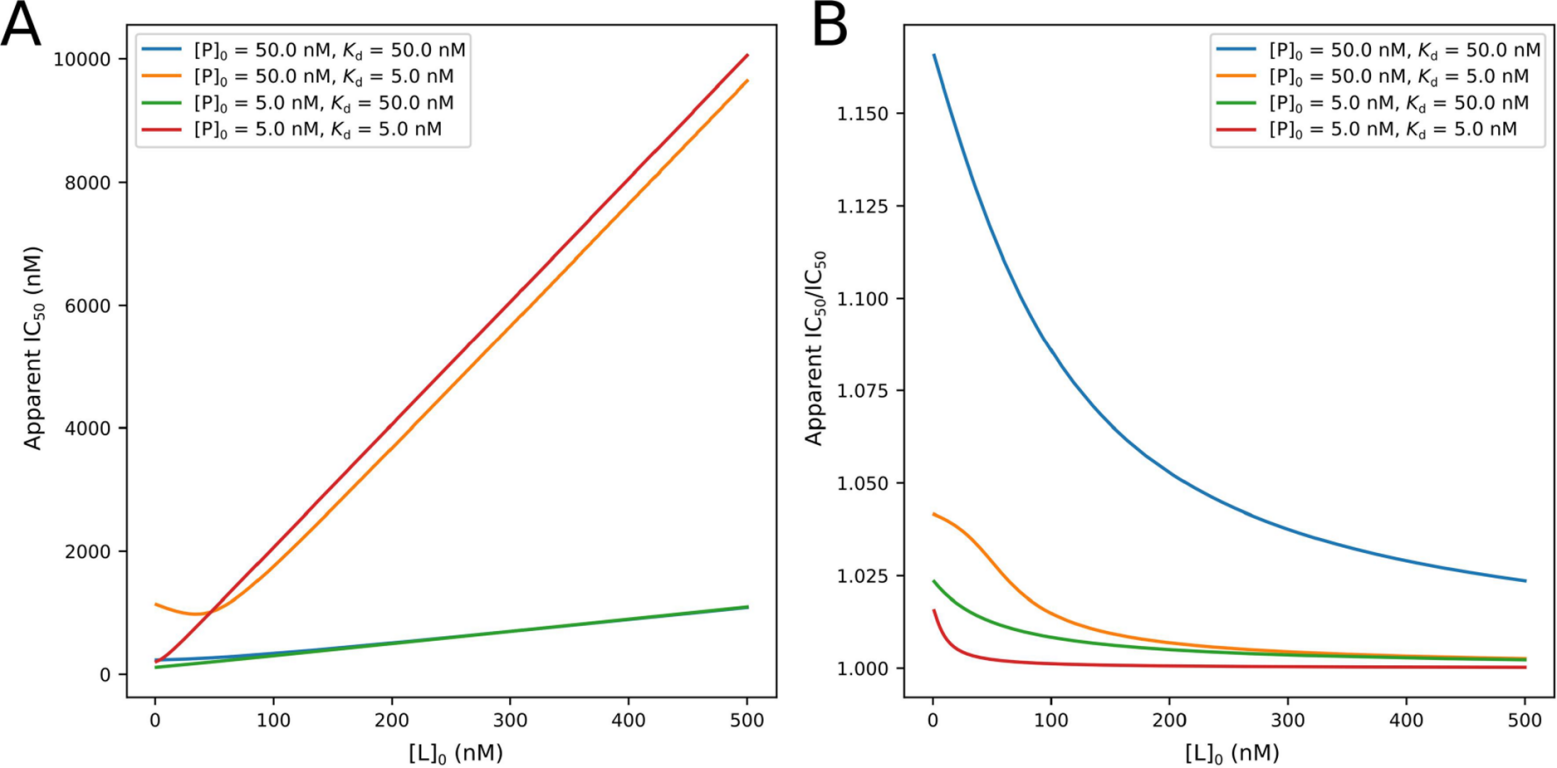
(A) Relationship between [L]_0_ and the apparent IC_50_. (B) Relationship of [L]_0_ and the ratio of the apparent IC_50_ to IC_50_ under different experimental conditions.

We plotted the theoretical inhibition curve and estimated IC_50_ and apparent IC_50_ under predefined conditions (Figure 2D). Researchers often use the Cheng–Prusoff equation^3^ to estimate *K*_i_ of the competitive inhibitor from the apparent IC_50_ in the condition of [L]_0_ ≫ [P]_0_ and [I]_0_ ≫ [P]_0_. Lin and Riggs^40^ also proposed an approach to estimate *K*_i_ in the condition of [L]_0_ ≈ [P]_0_ and [I]_0_ ≫ [P]_0_. In both approaches, the “[I]_0_ ≫ [P]_0_” condition essentially makes it possible to approximate [I]_eq_ by [I]_0_, i.e., the apparent IC_50_ is almost equal to IC_50_ (see Section 8 in SI for details) because the [I]_eq_ cannot be directly measured in most competitive binding experiments. Obviously, there are inherent errors in these approaches partly because IC_50_ and apparent IC_50_ may not be approximately the same under some conditions. For example, in exp. Nos. **10a** and **23a** (in Table S4), the difference between IC_50_ and apparent IC_50_ was greater than 2 and 5% of IC_50_, respectively. The “[P]_0_ > [L]_0_” condition of exp. No **23a** could be common in fluorescence polarization-based binding assays.^34^ We simulated the theoretical competitive binding curve with the inhibitor’s *K*_i_ being 100 nM in different experimental conditions by using the Wang equation^35^ to estimate IC_50_ and the apparent IC_50_. The results show that the larger the [L]_0_ value was, the larger apparent IC_50_ was needed to inhibit the binding of the protein and ligand (Figure 4A). Under the conditions of [P]_0_ = 50 nM and *K*_d_ = 5 nM and when [L]_0_ was lower than about 50 nM, most added inhibitors were depleted from the solution due to binding to the free protein. The lower [L]_0_ was, the more obvious the depletion of the inhibitor was. This explains why the apparent IC_50_ was higher under this condition. Although [P]_0_ had no obvious effect on the apparent IC_50_ under the condition of *K*_d_ = 50 nM (Figure 4A), the experiments with higher [P]_0_ showed a higher ratio of the apparent IC_50_ to IC_50_ (Figure 4B). The larger the *K*_d_ value was, the higher the ratio of the apparent IC_50_ to IC_50_ was. This suggests that to obtain a good approximation of IC_50_ by the apparent IC_50_, keeping [P]_0_ in a low range or choosing a ligand with a low *K*_d_ should be recommended.

**Figure 5:**
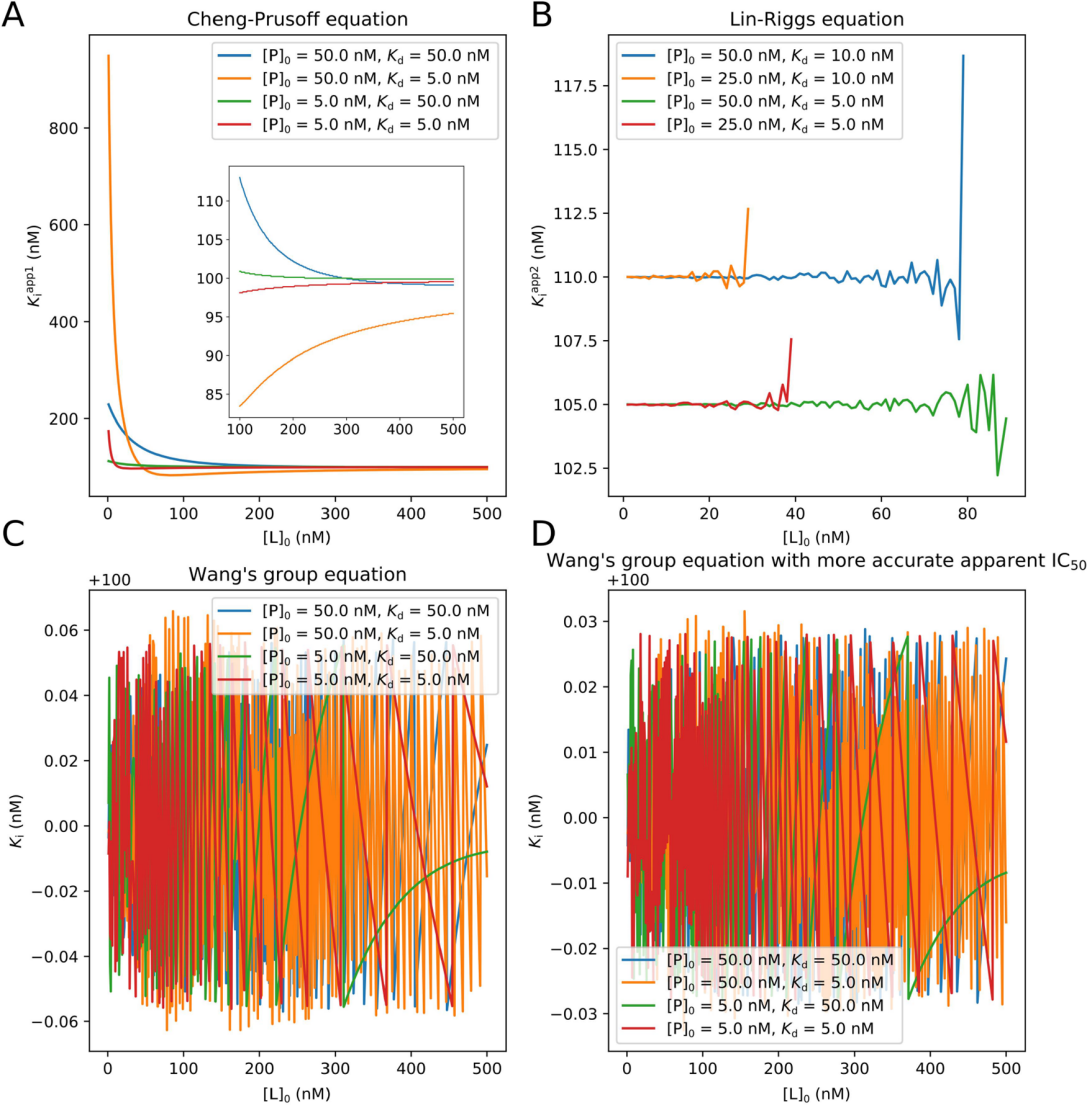
Under different experimental conditions, (A) relationship of the apparent *K*_i_ calculated by using the Cheng–Prusoff equation and [L]_0_, the relationship of the apparent *K*_i_ and [L]_0_ in the [L]_0_ range of 100 to 500 nM was inserted. (B) Relationship of the apparent *K*_i_ was calculated by using the Lin–Riggs equation and [L]_0_. The note about the experimental conditions and the range of [L]_0_ can be found in Section 8.2 in SI. (C) Relationship of *K*_i_ calculated by using Wang’s group equation and [L]_0_. (D) Relationship of *K*_i_ calculated by using the Wang’s group equation and [L]_0_ when more accurate apparent IC_50_ was used. The accuracy level of the estimation of the apparent IC_50_ was the same in (A), (B), and (C).

We further calculated *K*_i_ and the apparent *K*_i_ from the apparent IC_50_ by using three different equations. The theoretical inhibition curve was calculated by using the Wang equation^35^ in the condition of *K*_i_ constantly being 100 nM and [P]_0_ and *K*_d_ shown in the legends of Figure 5. Figure 5A shows that, by the Cheng–Prusoff equation, the larger the difference between [L]_0_ and [P]_0_ was, the more approximate the apparent *K*_i_ was to *K*_i_, which is consistent with the usage condition “[L]_0_ ≫ [P]_0_” of the Cheng–Prusoff equation. Figure 5B shows that when [L]_0_ ≈ [P]_0_ (i.e., [L]_0_ was in the range of [1, 80] nM), [P]_0_ = 50 nM, and *K*_d_ = 5 nM, the apparent *K*_i_ calculated by using the Lin–Riggs equation (the green line in Figure 5B) was more stable and accurate than the apparent *K*_i_ calculated by using the Cheng–Prusoff equation (the yellow line in Figure 5A), which is consistent with the usage condition “[L]_0_ ≈ [P]_0_” of the Lin–Riggs equation. In Figure 5B, the comparison of the conditions of *K*_d_ = 5 nM and *K*_d_ = 10 nM with [P]_0_ being constant reveals that the larger the *K*_d_ value was, the larger the apparent *K*_i_ was. The total concentration of the protein determined the permitted range of [L]_0_, i.e., the larger the [P]_0_ was, the wider the range of [L]_0_ was. Since Wang’s group derived the exact mathematical relationship of the apparent IC_50_ and *K*_i_ of the competitive inhibitor,^34^*K*_i_ can be calculated from the apparent IC_50_ without approximation as shown in Figure 5C,D. The calculated *K*_i_ and the theoretical *K*_i_ (100 nM) were in good agreement under different experimental conditions. The calculated *K*_i_ fluctuated in a smaller range (100 ± 0.03 vs 100 ± 0.06 nM) when more accurate apparent IC_50_ estimated by a finer simulation of the theoretical inhibition curve was used. This fluctuation came from the estimation process of the apparent IC_50_, which could not be eliminated or reduced by the calculation using a higher numerical precision (see Section 8.4 in the SI for details). Obviously, fitting the inhibition curve by using the Wang equation can give a direct estimation of *K*_i_, as researchers did in the fluorescence polarization^39,41^ and ITC^42^ experiments. We speculate that if there are enough measurements in the inhibition curve, especially in the end points, there should be no difference in the *K*_i_ values obtained by using Wang’s group equation and direct fitting. We will explore their differences in future work.

### Experimental Simulation of the High-Throughput Screening

In high-throughput screening, single measurements (*n* = 1) are usually employed in the initial primary screen because of the large number of samples to be tested. To theoretically access the sensitivity of the competitive binding assay in the primary screen, we conducted simulation experiments using the following default experimental conditions. The *K*_d_ value of the protein and labeled ligand was 100 nM. In experimental groups, after mixing, the initial working concentration of the inhibitor ([I]_0_) was 10 μM. The concentration of the total ligand ([L]_total_) was 10 nM. The equilibrium concentration of the protein–ligand complex in blank controls ([PL]_0_) ranged from 10 to 90% of [L]_total_.

**Figure 6:**
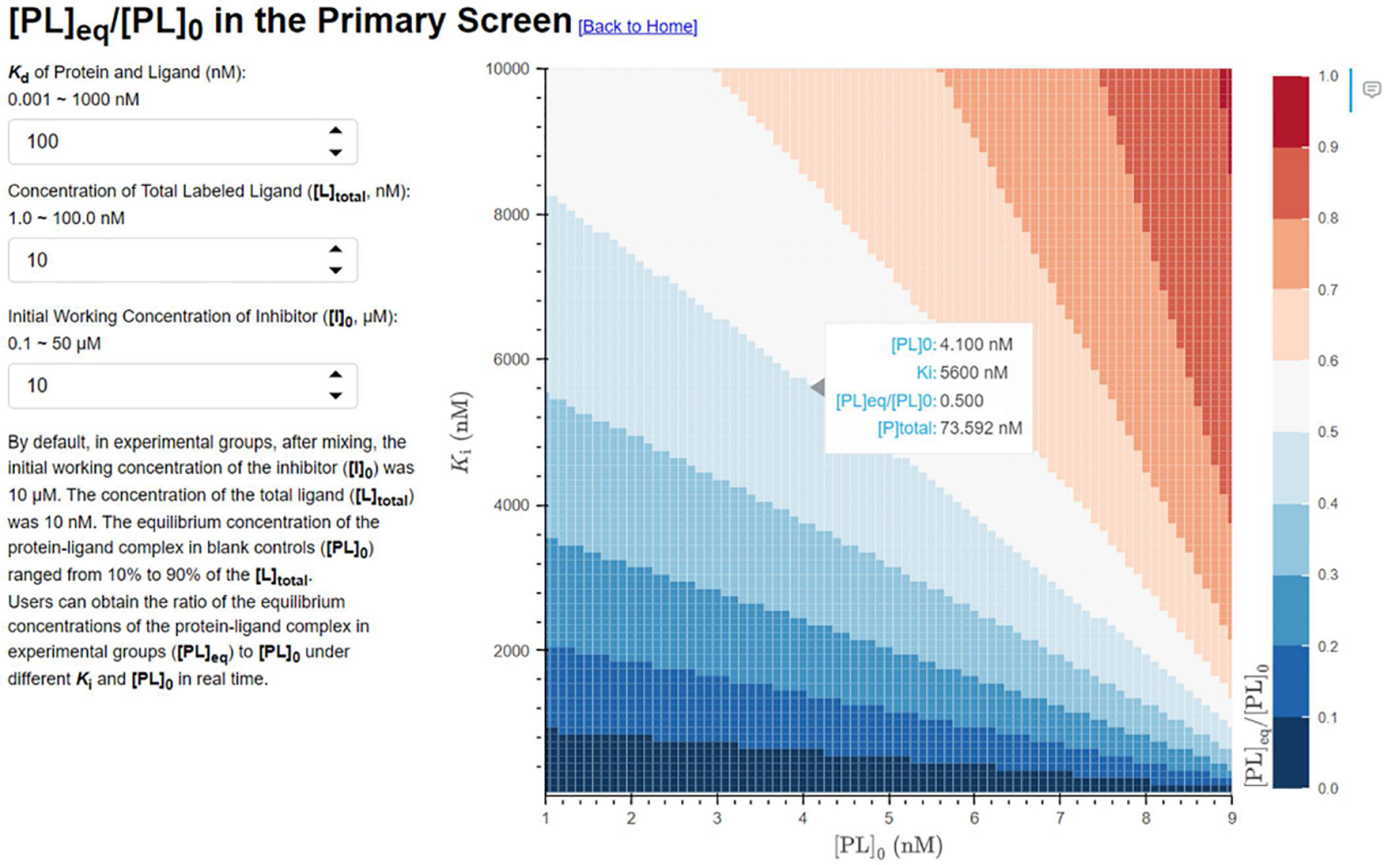
Screenshot of the functionality “[PL]_eq_/[PL]_0_ in the Primary Screen” in the Binding Curve Viewer. The hover displays related parameters on the graph.

Users can change the values of *K*_d_, [L]_total_, and [I]_0_ and obtain the ratio of the equilibrium concentrations of the protein–ligand complex in experimental groups ([PL]_eq_) to [PL]_0_ under different *K*_i_ and [PL]_0_ values in real time (Figure 6). Unlike concentration, researchers cannot adjust *K*_d_ and need to select one with a suitable *K*_d_ from a series of well-designed ligands. This tool can be used to visualize the [PL]_eq_/[PL]_0_ around a desired level of samples’ *K*_i_ under user-determined *K*_d_ and economical [P]_total_, [L]_total_, and [I]_0_. Ensuring sufficient signal change is also a critical factor in HTS experiments, so using low [PL] change out of the detection limit may not be proper. If [L]_total_ and [I]_0_ were fixed, we calculated [PL]_eq_/[PL]_0_ on the *K*_d_–*K*_i_ matrix (Figure S11) and observed results analogous to those reported in Shave et al.’s work.^30^ In contrast to the primary screen, to accurately calculate the apparent *K*_i_ using the Cheng–Prusoff equation, the apparent IC_50_ needs to be close to IC_50_ and the ligand with the smallest *K*_d_ is preferred (Figure S12). In addition to the above application in HTS simulation, we also used the Binding Curve Viewer to compare real experiments. See Section 11 in the SI for details.

## Conclusions

Both thermodynamic and kinetic properties are important metrics in the determination and optimization of protein–ligand interaction. In this work, we developed Binding Curve Viewer to analyze the thermodynamic and kinetic process of the protein–ligand binding and inhibitor’s competitive binding with 1:1 stoichiometry and no cooperativity. The theoretical saturation and kinetic curves were calculated or simulated accurately. The webpage of Binding Curve Viewer facilitated the comparison of *K*_d_ and apparent *K*_d_, the visualization of the kinetic experiment of association and dissociation, the visualization of the competitive binding, and the comparison of IC_50_ and apparent IC_50_. On the Web site of Binding Curve Viewer, users can input the thermodynamic and kinetic properties and the concentration of the protein, ligand, and inhibitor. The impact of parameter adjustments on the binding curves can be readily observed.

Using the program Binding Curve Viewer offers an opportunity to understand the binding process and assess the constraints imposed by different parameters on the estimation of the thermodynamic and kinetic properties. When fitting the data points from the theoretical kinetic curve to the pseudo-first-order binding process in the kinetic experiment, we found that there was a proper range of the concentration of the ligand to accurately estimate *k*_obs_, *k*_off_, and *k*_on_ at the same time. We showed the calculation of *K*_i_ by using the Cheng–Prusoff equation, Lin–Riggs equation, and Wang’s group equation under different conditions. Cheng–Prusoff equation and Lin–Riggs equation gave a proper estimation of *K*_i_ under their respective applicable conditions. The calculation of *K*_i_ by using Wang’s group equation outperformed the other two equations across all the experimental conditions we established. We also demonstrated the change in the concentration of the protein–ligand complex under different inhibition conditions in HTS experiments. For specific binding assays, it is still necessary to consider the method of experiments and the effect of the proportions of various species on the detection signal.

The web application and source code of Binding Curve Viewer are freely available on the Internet. By using them, we visualized the binding curves of the saturation experiment, kinetic experiment, and competition experiment and demonstrated the theoretical considerations in the estimation of the kinetic properties and inhibitory constant. We hope our tools can help researchers plan, interpret, and verify their interests in binding experiments. Our work will be one of the efforts to raise the community’s awareness of the overlooked parts in the binding experiments.

## Data Availability

The source code and web application of Binding Curve Viewer are freely available in https://github.com/ydu-sci/Binding_Curve_Viewer and https://eplatton.net/binding-curve-viewer, respectively.

## Supporting Information Available

Solutions of differential equations and equilibrium concentrations, prediction of the kinetic properties from the association kinetic experiment, examples of the iso-affinity graph, error analysis of the simulation of the competitive binding of association and dissociation, hypothetical association and dissociation kinetic experiments, hypothetical competitive binding experiments of association and dissociation, [PL] curves in “Competitive Binding Kinetics—Dissociation” showed S-shape in increasing concentrations of free protein, the calculation of *K*_i_ and apparent *K*_i_, protein and ligand with optimal *K*_d_ showed the highest sensitivity in the primary screen, competitive binding experiments under different conditions, and comparison with experimental results (PDF)
