# Supplementary material for: Binding Curve Viewer: Visualizing the Equilibrium and Kinetics of Protein–Ligand Binding and Competitive Binding
Source: J Chem Inf Model. 2024 May 8;64(10):4180–92. doi: 10.1021/acs.jcim.4c00130 (PMC11134506; doi:10.1021/acs.jcim.4c00130)
Supplement: Supplementary file 1 — ci4c00130_si_001.pdf [file ci4c00130_si_001.pdf]

Supporting Information for Binding Curve Viewer: Visualizing the Equilibrium and Kinetics of Protein-Ligand Binding and Competitive Binding

Yu Du

Department of Clinical Laboratory, The Second Affiliated Hospital of Jiaxing University, Huancheng North Road 1518, Jiaxing, Zhejiang, 314000, P. R. China

The Key Laboratory, The Second Affiliated Hospital of Jiaxing University, Huancheng North Road 1518, Jiaxing, Zhejiang, 314000, P. R. China

duyu@zjxu.edu.cn (Primary); ydu-sci@outlook.com

https://orcid.org/0000-0002-4114-396X

Table of Contents

1 Solutions of differential equations and equilibrium concentrations 3

1.1 Kinetics of the second-order binding process 3

1.2 Thermodynamics of the second-order binding process 4

1.3 Kinetics of the pseudo-first-order binding process 6

1.4 Thermodynamics of the pseudo-first-order binding process 7

1.5 Kinetics of the dissociation process 8

2 Prediction of the kinetic properties from the association kinetic experiment 9

3 Examples of the iso-affinity graph 14

4 Error analysis of the simulation of the competitive binding of association and dissociation 15

5 Hypothetical association and dissociation kinetic experiments 16

6 Hypothetical competitive binding experiments of association and dissociation 18

7 [PL] curves in "Competitive Binding Kinetics – Dissociation" showed S-shape in increasing concentrations of free protein 21

8 The calculation of the K_i and apparent K_i 22

8.1 Calculation of the apparent K_i by the Cheng-Prusoff equation 23

8.2 Calculation of the apparent K_i by the Lin-Riggs equation 24

8.3 Calculation of the K_i by the Wang's group equation 25

8.4 High-precision calculation of the K_i by the Wang's group equation 25

9 Protein and ligand with optimal K_d showed the highest sensitivity in primary screen 27

10 Competitive binding experiments under different conditions 29

11 Comparison with experimental results 30

Reference 34

1 Solutions of differential equations and equilibrium concentrations

Throughout this section, we describe the binding process initiated by mixing an arbitrary volume of protein with an arbitrary volume of ligand. This meant that there was no protein-ligand complex at the start of the binding process. The binding of the protein and ligand was 1:1 stoichiometry and had no cooperativity. In the dissociation process, we assumed that there was no rebinding.

1.1 Kinetics of the second-order binding process

The binding of the protein and ligand is described by the following equation,

$$\mathrm{P} + \mathrm{L} \underset{k_{\mathrm{off}}}{\overset{k_{\mathrm{on}}}{\rightleftharpoons}} \mathrm{PL}$$

The concentration of the protein-ligand complex changes during the binding process and

$$\frac{\mathrm{d[PL]}}{\mathrm{d}t} = k_{\mathrm{on}}[\mathrm{P}][\mathrm{L}] - k_{\mathrm{off}}[\mathrm{PL}] \quad \textbf{(1.1)}$$

where the [P] and [L] are the concentrations of the unbound protein and ligand, the k_{on} and k_{off} is respectively the association rate constant and dissociation rate constant. The concentration of the total protein and ligand, $[\mathrm{P}]_0$ and $[\mathrm{L}]_0$, and the concentration of the unbound protein and ligand, [P] and [L] are related by

$$[\mathrm{P}] = [\mathrm{P}]_0 - [\mathrm{PL}]$$

$$[\mathrm{L}] = [\mathrm{L}]_0 - [\mathrm{PL}]$$

Thus,

$$\frac{\mathrm{d[PL]}}{\mathrm{d}t} = k_{\mathrm{on}}([\mathrm{P}]_0 - [\mathrm{PL}])([\mathrm{L}]_0 - [\mathrm{PL}]) - k_{\mathrm{off}}[\mathrm{PL}]$$

$$\frac{\mathrm{d[PL]}}{\mathrm{d}t} = k_{\mathrm{on}}[\mathrm{PL}]^2 - (k_{\mathrm{on}}[\mathrm{P}]_0 + k_{\mathrm{on}}[\mathrm{L}]_0 + k_{\mathrm{off}})[\mathrm{PL}] + k_{\mathrm{on}}[\mathrm{P}]_0[\mathrm{L}]_0$$

$$\frac{\mathrm{d[PL]}}{k_{\mathrm{on}}\mathrm{d}t} = [\mathrm{PL}]^2 - \left([\mathrm{P}]_0 + [\mathrm{L}]_0 + \frac{k_{\mathrm{off}}}{k_{\mathrm{on}}}\right)[\mathrm{PL}] + [\mathrm{P}]_0[\mathrm{L}]_0 \quad \textbf{(1.2)}$$

The coefficients and discriminant of the right-hand side quadratic equation are

$$\mathrm{a} = 1, \mathrm{b} = -\left([\mathrm{P}]_0 + [\mathrm{L}]_0 + \frac{k_{\mathrm{off}}}{k_{\mathrm{on}}}\right) = -\left([\mathrm{P}]_0 + [\mathrm{L}]_0 + K_{\mathrm{d}}\right), \mathrm{c} = [\mathrm{P}]_0[\mathrm{L}]_0$$

$$\Delta = \mathrm{b}^2 - 4\mathrm{ac} = \left([\mathrm{P}]_0 + [\mathrm{L}]_0 + K_{\mathrm{d}}\right)^2 - 4[\mathrm{P}]_0[\mathrm{L}]_0 = \left([\mathrm{P}]_0 - [\mathrm{L}]_0\right)^2 + K_{\mathrm{d}}^{\,2} + 2[\mathrm{P}]_0 K_{\mathrm{d}} + 2[\mathrm{L}]_0 K_{\mathrm{d}} > 0$$

Thus, two distinct real roots are

$$[PL]_1 = \frac{-b - \sqrt{b^2 - 4ac}}{2a} = \frac{\left([P]_0 + [L]_0 + K_d\right) - \sqrt{\left([P]_0 + [L]_0 + K_d\right)^2 - 4[P]_0[L]_0}}{2}$$

$$[PL]_2 = \frac{-b + \sqrt{b^2 - 4ac}}{2a}$$

Then equation 1.2 may be written as

$$\frac{\mathrm{d}[\mathrm{PL}]}{k_{\mathrm{on}}\mathrm{d}t} = ([\mathrm{PL}] - [\mathrm{PL}]_1)([\mathrm{PL}] - [\mathrm{PL}]_2)$$

$$\frac{\mathrm{d}[\mathrm{PL}]}{([\mathrm{PL}] - [\mathrm{PL}]_1)([\mathrm{PL}] - [\mathrm{PL}]_2)} = k_{\mathrm{on}}\mathrm{d}t$$

$$\frac{1}{[\mathrm{PL}]_1 - [\mathrm{PL}]_2}\left(\frac{1}{[\mathrm{PL}] - [\mathrm{PL}]_1} - \frac{1}{[\mathrm{PL}] - [\mathrm{PL}]_2}\right)\mathrm{d}[\mathrm{PL}] = k_{\mathrm{on}}\mathrm{d}t$$

$$\frac{\mathrm{d}[\mathrm{PL}]}{[\mathrm{PL}] - [\mathrm{PL}]_1} - \frac{\mathrm{d}[\mathrm{PL}]}{[\mathrm{PL}] - [\mathrm{PL}]_2} = k_{\mathrm{on}}([\mathrm{PL}]_1 - [\mathrm{PL}]_2)\mathrm{d}t$$

$$\ln\left|[\mathrm{PL}] - [\mathrm{PL}]_1\right| + \mathrm{C}_1 - \ln\left|[\mathrm{PL}] - [\mathrm{PL}]_2\right| + \mathrm{C}_2 = k_{\mathrm{on}}\left([\mathrm{PL}]_1 - [\mathrm{PL}]_2\right)t + \mathrm{C}_3$$

Because $\mathrm{b} < 0$, $[\mathrm{PL}]_1 < [\mathrm{PL}]_2$. From the expression of $[\mathrm{PL}]_1$, we know that $[\mathrm{PL}]_1 > 0$. When [PL] increases from 0 to $[\mathrm{PL}]_1$, $\frac{\mathrm{d}[\mathrm{PL}]}{\mathrm{d}t}$ decreases from the maximum value to 0, i.e., $\frac{\mathrm{d}[\mathrm{PL}]}{\mathrm{d}t} \to 0$, the binding process approaches equilibrium, so $[\mathrm{PL}] \in \left[0, [\mathrm{PL}]_1\right)$. Thus, the above equation gives

$$\ln\left([\mathrm{PL}]_1 - [\mathrm{PL}]\right) - \ln\left([\mathrm{PL}]_2 - [\mathrm{PL}]\right) = k_{\mathrm{on}}\left([\mathrm{PL}]_1 - [\mathrm{PL}]_2\right)t + \mathrm{C}_4$$

When $t = 0$, $[\mathrm{PL}] = 0$. Thus

$$\ln\left([\mathrm{PL}]_1 - 0\right) - \ln\left([\mathrm{PL}]_2 - 0\right) = k_{\mathrm{on}}\left([\mathrm{PL}]_1 - [\mathrm{PL}]_2\right) \times 0 + \mathrm{C}_4$$

$C_4 = \ln\frac{[PL]_1}{[PL]_2}$, substituting the above equation gives

$$\ln\left([\mathrm{PL}]_1 - [\mathrm{PL}]\right) - \ln\left([\mathrm{PL}]_2 - [\mathrm{PL}]\right) = k_{\mathrm{on}}\left([\mathrm{PL}]_1 - [\mathrm{PL}]_2\right)t + \ln\frac{[\mathrm{PL}]_1}{[\mathrm{PL}]_2}$$

$$t = \frac{1}{k_{\mathrm{on}}\left([\mathrm{PL}]_1 - [\mathrm{PL}]_2\right)}\ln\frac{[\mathrm{PL}]_2([\mathrm{PL}]_1 - [\mathrm{PL}])}{[\mathrm{PL}]_1([\mathrm{PL}]_2 - [\mathrm{PL}])} \quad \textbf{(1.3)}$$

1.2 Thermodynamics of the second-order binding process

The equilibrium state of the binding process is described by the following equation,

$$K_{\mathrm{d}} = \frac{[\mathrm{P}]_{\mathrm{eq}}[\mathrm{L}]_{\mathrm{eq}}}{[\mathrm{PL}]_{\mathrm{eq}}}$$

where the $[\mathrm{P}]_{\mathrm{eq}}$, $[\mathrm{L}]_{\mathrm{eq}}$, and $[\mathrm{PL}]_{\mathrm{eq}}$ is the equilibrium concentration of the unbound protein and ligand and the equilibrium concentration of the protein-ligand complex. The total protein and ligand concentration, $[\mathrm{P}]_0$ and $[\mathrm{L}]_0$, and the equilibrium concentration of the unbound protein and ligand $[\mathrm{P}]_{\mathrm{eq}}$ and $[\mathrm{L}]_{\mathrm{eq}}$ are related by

$$[\mathrm{P}]_{\mathrm{eq}} = [\mathrm{P}]_0 - [\mathrm{PL}]_{\mathrm{eq}}$$

$$[\mathrm{L}]_{\mathrm{eq}} = [\mathrm{L}]_0 - [\mathrm{PL}]_{\mathrm{eq}}$$

Thus,

$$K_{\mathrm{d}} = \frac{([\mathrm{P}]_0 - [\mathrm{PL}]_{\mathrm{eq}})([\mathrm{L}]_0 - [\mathrm{PL}]_{\mathrm{eq}})}{[\mathrm{PL}]_{\mathrm{eq}}}$$

$$K_{\mathrm{d}}[\mathrm{PL}]_{\mathrm{eq}} = ([\mathrm{P}]_0 - [\mathrm{PL}]_{\mathrm{eq}})([\mathrm{L}]_0 - [\mathrm{PL}]_{\mathrm{eq}})$$

$$([\mathrm{PL}]_{\mathrm{eq}})^2 - \left([\mathrm{P}]_0 + [\mathrm{L}]_0 - K_{\mathrm{d}}\right)[\mathrm{PL}]_{\mathrm{eq}} + [\mathrm{P}]_0[\mathrm{L}]_0 = 0 \qquad \textbf{(1.4)}$$

This equation is the same as the right-hand side of equation 1.2, its coefficients and discriminant are

$$\mathrm{a} = 1, \mathrm{b} = -\left([\mathrm{P}]_0 + [\mathrm{L}]_0 + \frac{k_{\mathrm{off}}}{k_{\mathrm{on}}}\right) = -\left([\mathrm{P}]_0 + [\mathrm{L}]_0 + K_{\mathrm{d}}\right), \mathrm{c} = [\mathrm{P}]_0[\mathrm{L}]_0$$

$$\Delta = \mathrm{b}^2 - 4\mathrm{ac} = \left([\mathrm{P}]_0 + [\mathrm{L}]_0 + K_{\mathrm{d}}\right)^2 - 4[\mathrm{P}]_0[\mathrm{L}]_0 = \left([\mathrm{P}]_0 - [\mathrm{L}]_0\right)^2 + {K_{\mathrm{d}}}^2 + 2[\mathrm{P}]_0K_{\mathrm{d}} + 2[\mathrm{L}]_0K_{\mathrm{d}} > 0$$

$$[\mathrm{PL}]_{\mathrm{eq1}} = \frac{-\mathrm{b} - \sqrt{\mathrm{b}^2 - 4\mathrm{ac}}}{2\mathrm{a}} = \frac{\left([\mathrm{P}]_0 + [\mathrm{L}]_0 + K_{\mathrm{d}}\right) - \sqrt{\left([\mathrm{P}]_0 + [\mathrm{L}]_0 + K_{\mathrm{d}}\right)^2 - 4[\mathrm{P}]_0[\mathrm{L}]_0}}{2} \qquad \textbf{(1.5)}$$

$$[\mathrm{PL}]_{\mathrm{eq2}} = \frac{-\mathrm{b} + \sqrt{\mathrm{b}^2 - 4\mathrm{ac}}}{2\mathrm{a}} = \frac{\left([\mathrm{P}]_0 + [\mathrm{L}]_0 + K_{\mathrm{d}}\right) + \sqrt{\left([\mathrm{P}]_0 + [\mathrm{L}]_0 + K_{\mathrm{d}}\right)^2 - 4[\mathrm{P}]_0[\mathrm{L}]_0}}{2}$$

From the expressions of $[\mathrm{PL}]_{\mathrm{eq1}}$ and $[\mathrm{PL}]_{\mathrm{eq2}}$, we know that $0 > [\mathrm{PL}]_{\mathrm{eq1}} > [\mathrm{PL}]_{\mathrm{eq2}}$. We assume that the [PL] increases from 0 to the first real root to make the quadratic equation zero. So, $[\mathrm{PL}]_{\mathrm{eq1}}$ should be the equilibrium concentration of the protein-ligand complex. It should be noted that the thermodynamic process of the binding reaction is independent of the initial concentrations of the binding species. Hence, we can calculate the total concentration of the protein and ligand from the initial binding system and use the approaches presented here to calculate the equilibrium concentration of the protein, ligand, and protein-ligand complex.

1.3 Kinetics of the pseudo-first-order binding process

The binding of the protein and ligand is described by the following equation,

$$\mathrm{P} + \mathrm{L} \underset{k_{\mathrm{off}}}{\overset{k_{\mathrm{on}}}{\rightleftharpoons}} \mathrm{PL}$$

If $[\mathrm{L}]_0 \gg [\mathrm{P}]_0$, the binding process is effectively first-order since the [L] is hardly affected by the binding process, then the equation can be transformed to

$$\mathrm{P} \underset{k_{\mathrm{off}}}{\overset{k_{\mathrm{on}} \times [\mathrm{L}]_0}{\rightleftharpoons}} \mathrm{PL}$$

The concentration of the protein-ligand complex changes during the binding process and

$$\frac{\mathrm{d}[\mathrm{PL}]}{\mathrm{d}t} = k_{\mathrm{on}}[\mathrm{P}][\mathrm{L}] - k_{\mathrm{off}}[\mathrm{PL}]$$

During the binding process, $[\mathrm{L}] \approx [\mathrm{L}]_0$, then

$$\frac{\mathrm{d}[\mathrm{PL}]}{\mathrm{d}t} = k_{\mathrm{on}}[\mathrm{P}][\mathrm{L}]_0 - k_{\mathrm{off}}[\mathrm{PL}]$$

The total protein concentration ($[\mathrm{P}]_0$) and the concentration of the unbound protein ([P]) are related by

$$[\mathrm{P}] = [\mathrm{P}]_0 - [\mathrm{PL}]$$

Thus,

$$\frac{\mathrm{d}[\mathrm{PL}]}{\mathrm{d}t} = k_{\mathrm{on}}[\mathrm{L}]_0([\mathrm{P}]_0 - [\mathrm{PL}]) - k_{\mathrm{off}}[\mathrm{PL}]$$

When [PL] increases from 0 to $[\mathrm{PL}]_{\mathrm{eq}}$, $\frac{\mathrm{d}[\mathrm{PL}]}{\mathrm{d}t}$ decreases from the maximum value to 0. $\frac{\mathrm{d}[\mathrm{PL}]}{\mathrm{d}t} \rightarrow 0$ and $\frac{\mathrm{d}[\mathrm{PL}]}{\mathrm{d}t} > 0$,

$$\frac{\mathrm{d}[\mathrm{PL}]}{\mathrm{d}t} = k_{\mathrm{on}}[\mathrm{L}]_0[\mathrm{P}]_0 - (k_{\mathrm{on}}[\mathrm{L}]_0 + k_{\mathrm{off}})[\mathrm{PL}] > 0 \quad \textbf{(1.6)}$$

$$\frac{\mathrm{d}\{k_{\mathrm{on}}[\mathrm{L}]_0[\mathrm{P}]_0 - (k_{\mathrm{on}}[\mathrm{L}]_0 + k_{\mathrm{off}})[\mathrm{PL}]\}}{k_{\mathrm{on}}[\mathrm{L}]_0[\mathrm{P}]_0 - (k_{\mathrm{on}}[\mathrm{L}]_0 + k_{\mathrm{off}})[\mathrm{PL}]} = -(k_{\mathrm{on}}[\mathrm{L}]_0 + k_{\mathrm{off}})\mathrm{d}t$$

$$\ln\left|k_{\mathrm{on}}[\mathrm{L}]_0[\mathrm{P}]_0 - (k_{\mathrm{on}}[\mathrm{L}]_0 + k_{\mathrm{off}})[\mathrm{PL}]\right| + \mathrm{C}_1 = -(k_{\mathrm{on}}[\mathrm{L}]_0 + k_{\mathrm{off}})t + \mathrm{C}_2$$

Because the right-hand side of equation 1.6 is greater than 0,

$$\ln\{k_{\mathrm{on}}[\mathrm{L}]_0[\mathrm{P}]_0 - (k_{\mathrm{on}}[\mathrm{L}]_0 + k_{\mathrm{off}})[\mathrm{PL}]\} = -(k_{\mathrm{on}}[\mathrm{L}]_0 + k_{\mathrm{off}})t + \mathrm{C}_3$$

When $t = 0$, $[\mathrm{PL}] = 0$. Thus, $\mathrm{C}_3 = \ln(k_{\mathrm{on}}[\mathrm{L}]_0[\mathrm{P}]_0)$, substituting the above equation gives

$$\ln\{k_{\mathrm{on}}[\mathrm{L}]_0[\mathrm{P}]_0 - (k_{\mathrm{on}}[\mathrm{L}]_0 + k_{\mathrm{off}})[\mathrm{PL}]\} = -(k_{\mathrm{on}}[\mathrm{L}]_0 + k_{\mathrm{off}})t + \ln(k_{\mathrm{on}}[\mathrm{L}]_0[\mathrm{P}]_0) \quad \textbf{(1.7)}$$

$$[\mathrm{PL}] = \frac{k_{\mathrm{on}}[\mathrm{L}]_0[\mathrm{P}]_0}{k_{\mathrm{on}}[\mathrm{L}]_0 + k_{\mathrm{off}}}(1 - e^{-(k_{\mathrm{on}}[\mathrm{L}]_0 + k_{\mathrm{off}})t})$$

When $t \to +\infty$,

$$[\mathrm{PL}]_{\mathrm{eq}} = \frac{k_{\mathrm{on}}[\mathrm{L}]_0[\mathrm{P}]_0}{k_{\mathrm{on}}[\mathrm{L}]_0 + k_{\mathrm{off}}} = \frac{[\mathrm{L}]_0[\mathrm{P}]_0}{[\mathrm{L}]_0 + K_{\mathrm{d}}} \quad \textbf{(1.8)}$$

Substitute equation 1.8 with equation 1.7 gives

$$[\mathrm{PL}] = [\mathrm{PL}]_{\mathrm{eq}}(1 - e^{-(k_{\mathrm{on}}[\mathrm{L}]_0 + k_{\mathrm{off}})t}) \quad \textbf{(1.9)}$$

Define the observation rate constant k_{obs} by

$$k_{\mathrm{obs}} = k_{\mathrm{on}}[\mathrm{L}]_0 + k_{\mathrm{off}} \quad \textbf{(1.10)}$$

Thus,

$$[\mathrm{PL}] = [\mathrm{PL}]_{\mathrm{eq}}(1 - e^{-k_{\mathrm{obs}}t}) \quad \textbf{(1.11)}$$

Transform equation 1.7 to

$$\ln\frac{k_{\mathrm{on}}[\mathrm{L}]_0[\mathrm{P}]_0 - (k_{\mathrm{on}}[\mathrm{L}]_0 + k_{\mathrm{off}})[\mathrm{PL}]}{k_{\mathrm{on}}[\mathrm{L}]_0[\mathrm{P}]_0} = -(k_{\mathrm{on}}[\mathrm{L}]_0 + k_{\mathrm{off}})t$$

$$t = \frac{-1}{k_{\mathrm{on}}[\mathrm{L}]_0 + k_{\mathrm{off}}}\ln(1 - \frac{(k_{\mathrm{on}}[\mathrm{L}]_0 + k_{\mathrm{off}})[\mathrm{PL}]}{k_{\mathrm{on}}[\mathrm{L}]_0[\mathrm{P}]_0}) \quad \textbf{(1.12)}$$

1.4 Thermodynamics of the pseudo-first-order binding process

The equilibrium state of the binding process is described by the following equation,

$$K_{\mathrm{d}} = \frac{[\mathrm{P}]_{\mathrm{eq}}[\mathrm{L}]_{\mathrm{eq}}}{[\mathrm{PL}]_{\mathrm{eq}}}$$

where $[\mathrm{P}]_{\mathrm{eq}}$, $[\mathrm{L}]_{\mathrm{eq}}$, and $[\mathrm{PL}]_{\mathrm{eq}}$ are the equilibrium concentrations of the unbound protein and ligand and the equilibrium concentration of the protein-ligand complex. If $[\mathrm{L}]_0 \gg [\mathrm{P}]_0$, or strictly speaking, $[\mathrm{L}]_0 \gg [\mathrm{PL}]_{\mathrm{eq}}$, $[\mathrm{L}]_{\mathrm{eq}} = [\mathrm{L}]_0 - [\mathrm{PL}]_{\mathrm{eq}} \approx [\mathrm{L}]_0$. Hence, the $[\mathrm{L}]_0$ is hardly affected by the binding process, then the equation can be transformed to

$$K_{\mathrm{d}} = \frac{[\mathrm{P}]_{\mathrm{eq}}[\mathrm{L}]_0}{[\mathrm{PL}]_{\mathrm{eq}}}$$

The total protein concentration ($[\mathrm{P}]_0$) and the equilibrium concentration of the unbound protein ($[\mathrm{P}]_{\mathrm{eq}}$) are related by

$$[\mathrm{P}]_{\mathrm{eq}} = [\mathrm{P}]_0 - [\mathrm{PL}]_{\mathrm{eq}}$$

Thus,

$$K_{\mathrm{d}} = \frac{([\mathrm{P}]_0 - [\mathrm{PL}]_{\mathrm{eq}})[\mathrm{L}]_0}{[\mathrm{PL}]_{\mathrm{eq}}}$$

$$K_{\mathrm{d}}[\mathrm{PL}]_{\mathrm{eq}} = ([\mathrm{P}]_0 - [\mathrm{PL}]_{\mathrm{eq}})[\mathrm{L}]_0$$

$$[\mathrm{PL}]_{\mathrm{eq}} = \frac{[\mathrm{L}]_0[\mathrm{P}]_0}{[\mathrm{L}]_0 + K_{\mathrm{d}}} \quad \textbf{(1.13)}$$

In either the second-order binding process or the pseudo-first-order binding process, the $[\mathrm{PL}]_{\mathrm{eq}}$ is the same calculated by the thermodynamic and kinetic approaches. But the equations calculated from the second-order binding process are different from the equations calculated from the pseudo-first-order binding process.

1.5 Kinetics of the dissociation process

The dissociation of the protein-ligand complex with no rebinding is described by the following equation,

$$\mathrm{PL} \xrightarrow{k_{\mathrm{off}}} \mathrm{P} + \mathrm{L}$$

The concentration of the protein-ligand complex changes during the dissociation process and

$$\frac{\mathrm{d}[\mathrm{PL}]}{\mathrm{d}t} = -\,k_{\mathrm{off}}[\mathrm{PL}]$$

$$\frac{\mathrm{d}[\mathrm{PL}]}{[\mathrm{PL}]} = -\,k_{\mathrm{off}}\mathrm{d}t$$

$$\ln|[\mathrm{PL}]| = -\,k_{\mathrm{off}}t + \mathrm{C}$$

$$\ln[\mathrm{PL}] = -\,k_{\mathrm{off}}t + \mathrm{C}$$

When $t = 0$, $[\mathrm{PL}] = [\mathrm{PL}]_0$. Thus, $\mathrm{C} = \ln[\mathrm{PL}]_0$, substituting the above equation gives

$$\ln[\mathrm{PL}] = -\,k_{\mathrm{off}}t + \ln[\mathrm{PL}]_0$$

$$t = -\frac{1}{k_{\mathrm{off}}}\ln\frac{[\mathrm{PL}]}{[\mathrm{PL}]_0} \quad \textbf{(1.14)}$$

2 Prediction of the kinetic properties from the association kinetic experiment

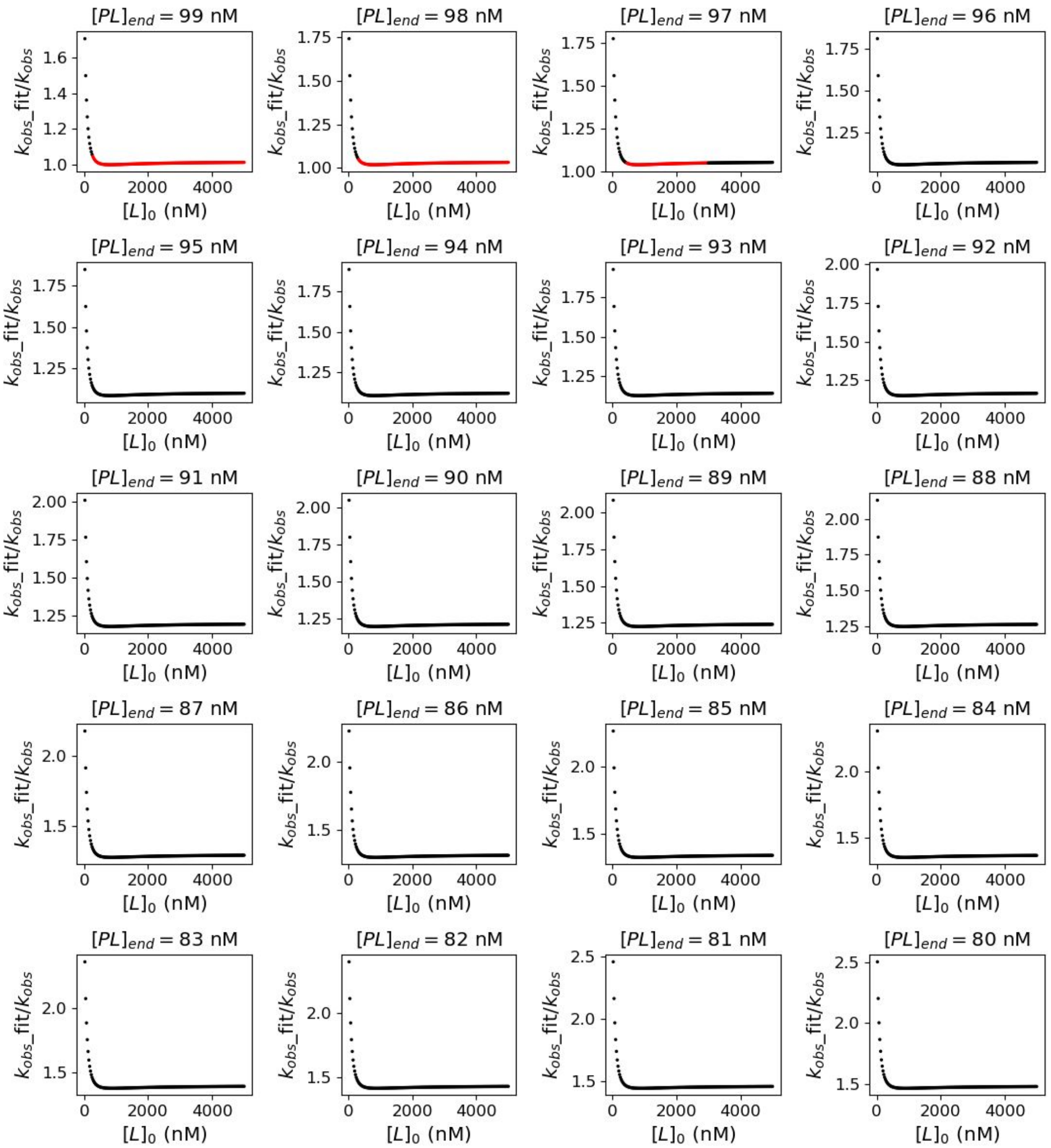

Figure S1. The relationship between k_{obs}_fit/k_{obs} and $[L]_0$ in the condition of $[P]_0$ = 100 nM, k_{off} = 0.01 s^{-1}, k_{on} = 1e5 M^{-1} s^{-1} and in different pre-equilibrium termination states of the kinetic experiments.

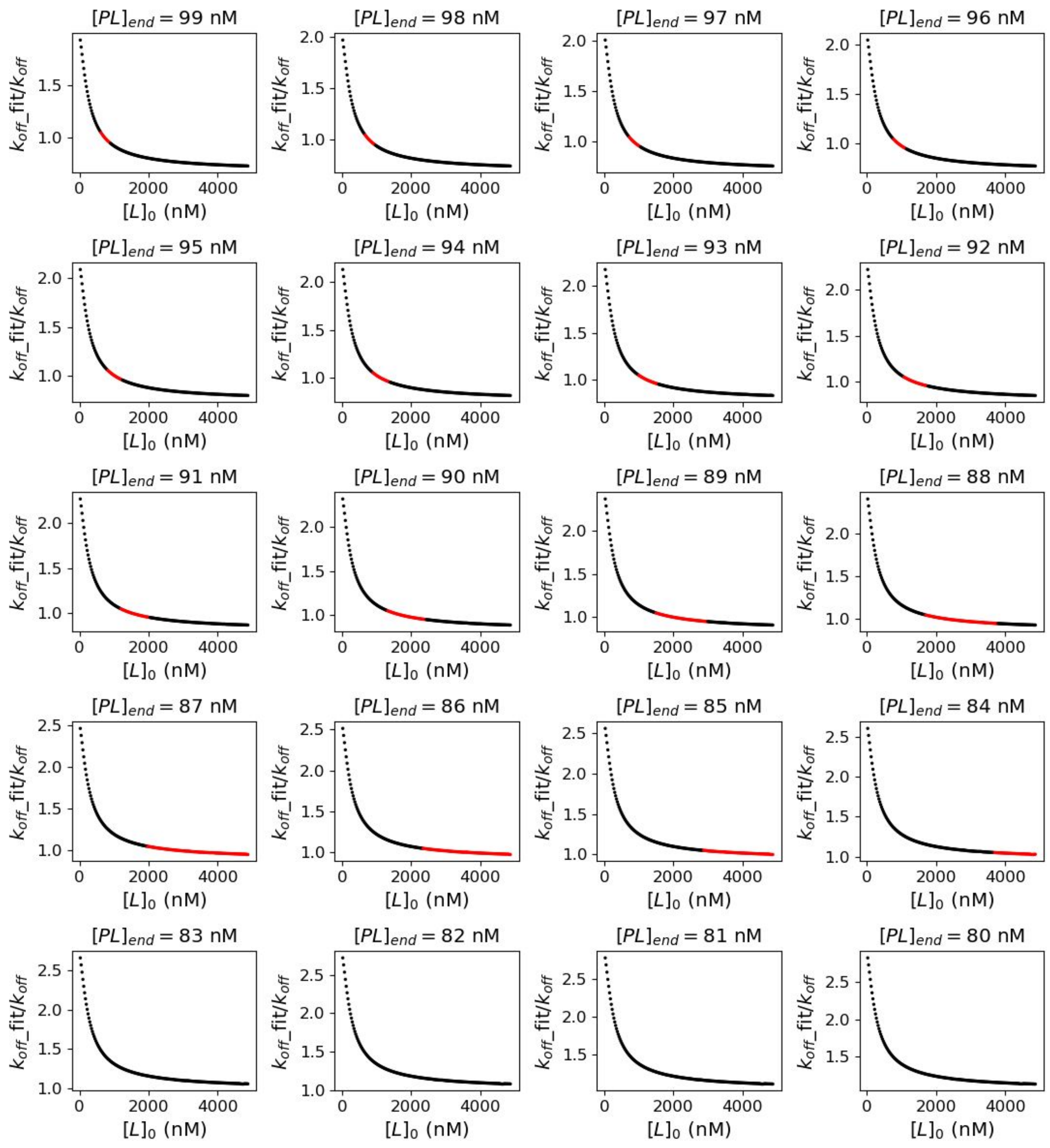

Figure S2. The relationship between k_{off}_fit/k_{off} and $[L]_0$ in the condition of $[P]_0$ = 100 nM, k_{off} = 0.01 s^{-1}, k_{on} = 1e5 M^{-1} s^{-1} and in different pre-equilibrium termination states of the kinetic experiments.

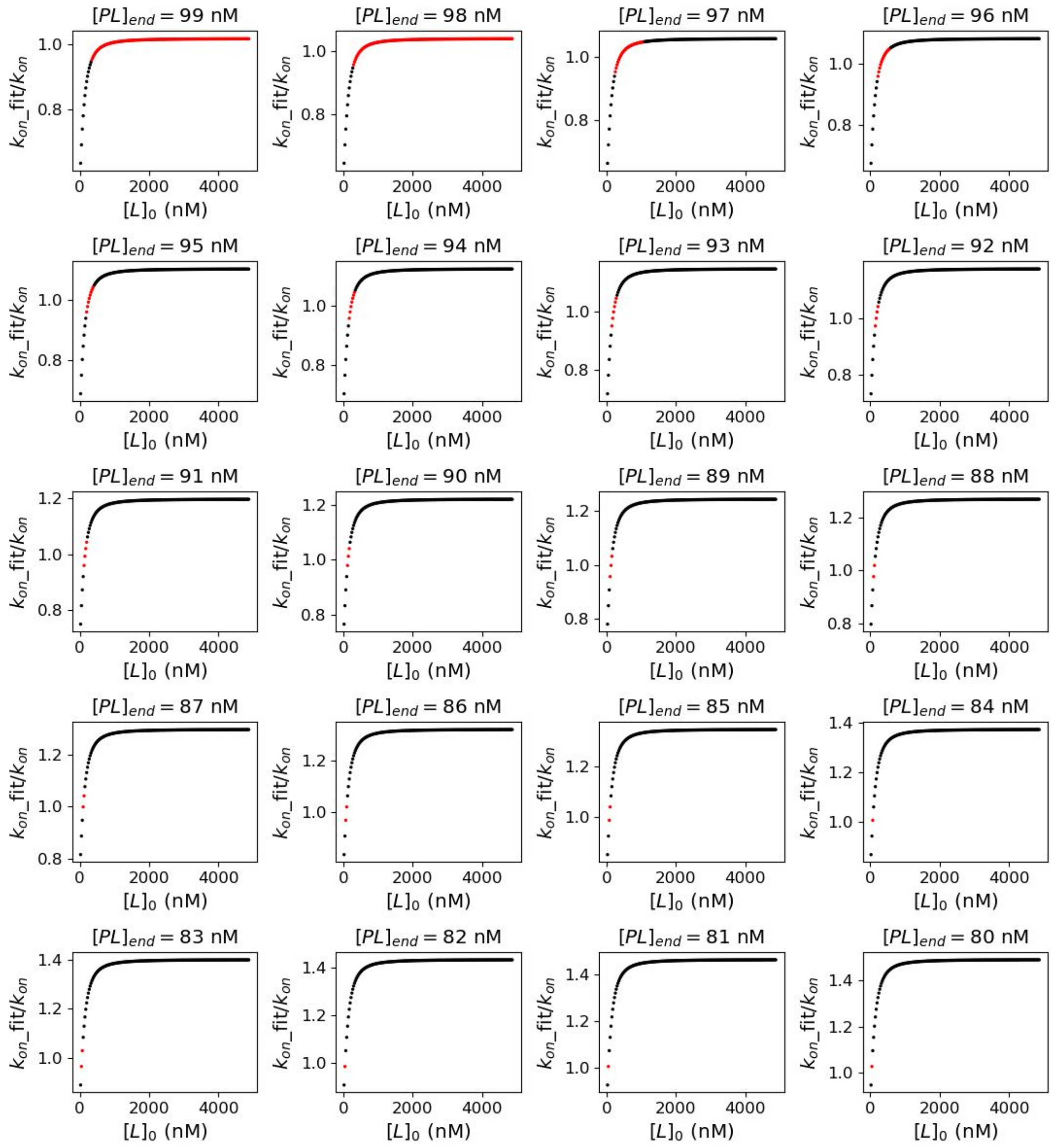

Figure S3. The relationship between k_{on}_fit/k_{on} and $[\mathrm{L}]_0$ in the condition of $[\mathrm{P}]_0$ = 100 nM, k_{off} = 0.01 s^{-1}, k_{on} = 1e5 M^{-1} s^{-1} and in different pre-equilibrium termination states of the kinetic experiments.

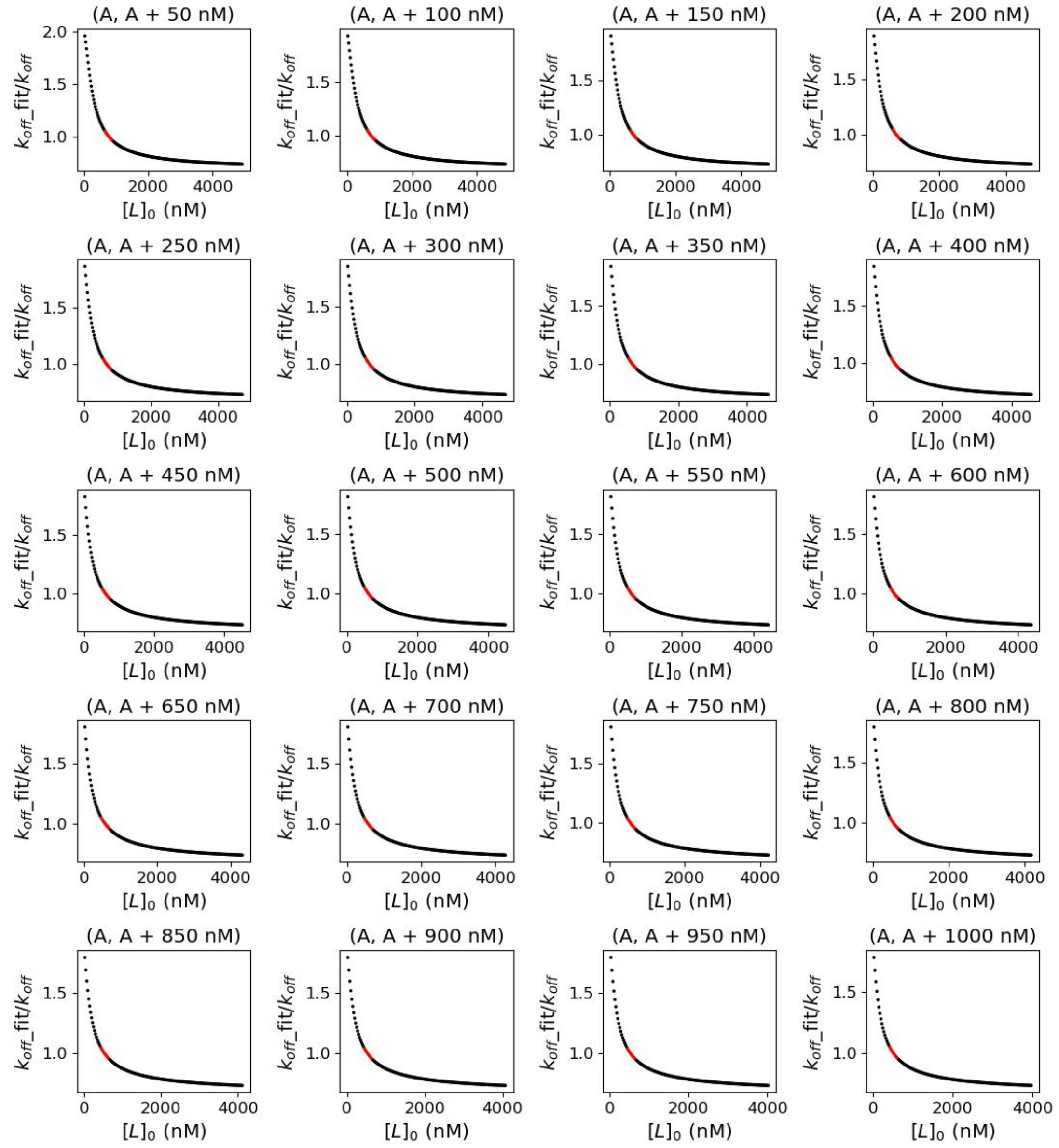

Figure S4. The relationship between k_{off}_fit/k_{off} and $[\mathrm{L}]_0$ in the condition of $[\mathrm{P}]_0$ = 100 nM, k_{off} = 0.01 s^{-1}, k_{on} = 1e5 M^{-1} s^{-1} and in different $[\mathrm{L}]_0$ between two measurements of the kinetic experiments. The end-point measurement was 99% of the equilibrium.

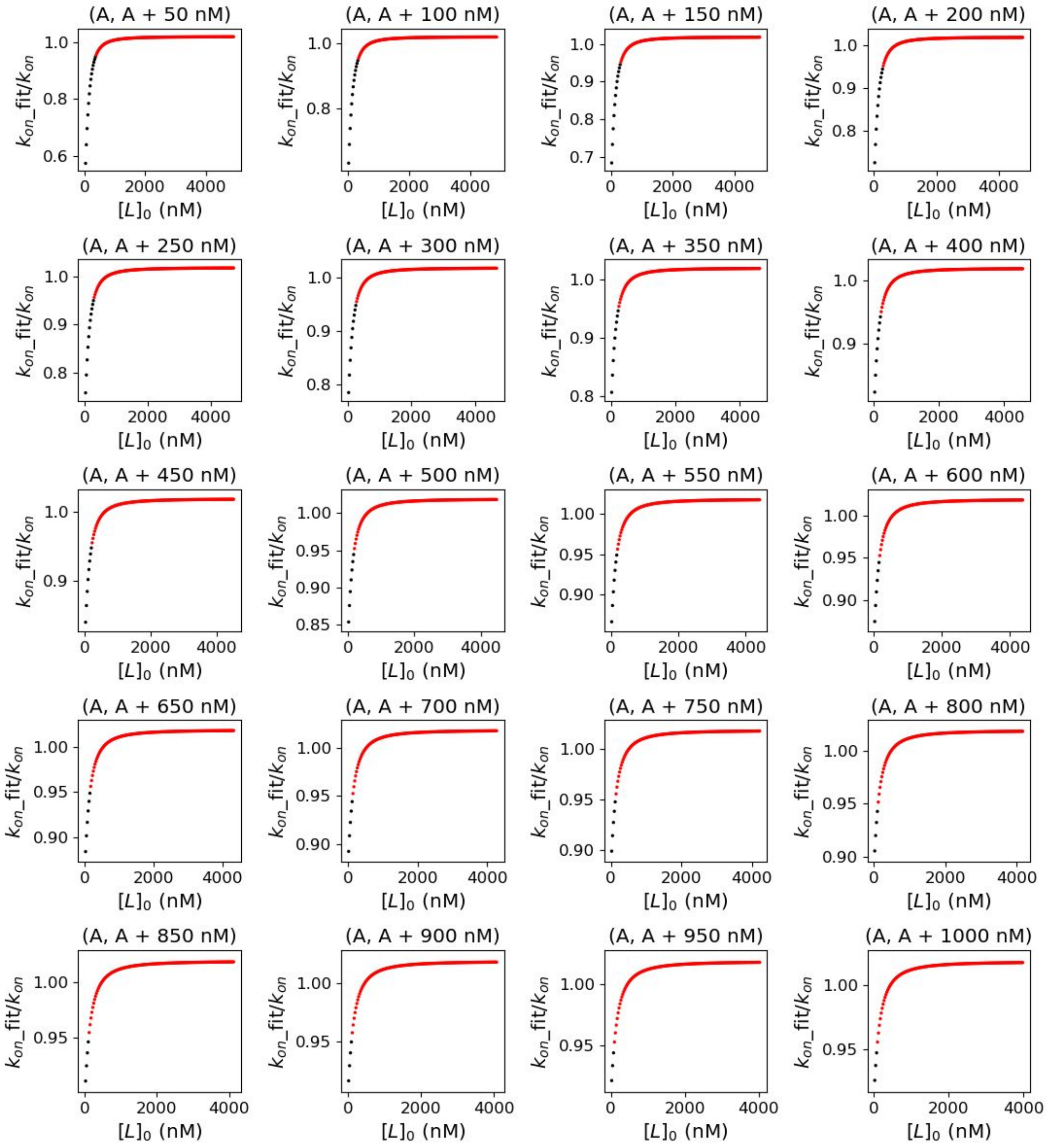

Figure S5. The relationship between k_{on}_fit/k_{on} and $[L]_0$ in the condition of $[P]_0$ = 100 nM, k_{off} = 0.01 s^{-1}, k_{on} = 1e5 M^{-1} s^{-1} and in different $[L]_0$ between two measurements of the kinetic experiments. The end-point measurement was 99% of the equilibrium.

3 Examples of the iso-affinity graph

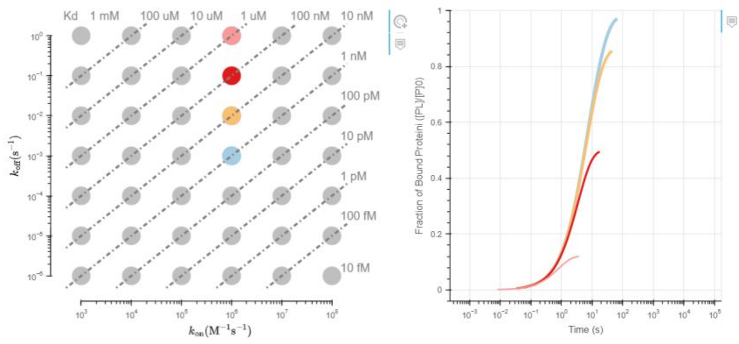

The time to 50% and 99% equilibrium and **[PL]eq** under specific initial conditions (**[P]0** = 100 nM and **[L]0** = 150 nM) and different kinetic parameters in the second-order binding process:

#	Kd (M)	koff (s-1)	kon (M-1 s-1)	[PL]eq (nM)	t0.50 (s)	t0.99 (s)
21	1.00e-09	1.00e-03	1.00e+06	9.81e+01	5.59e+00	6.57e+01
27	1.00e-08	1.00e-02	1.00e+06	8.64e+01	4.67e+00	4.50e+01
33	1.00e-07	1.00e-01	1.00e+06	5.00e+01	2.42e+00	1.77e+01
39	1.00e-06	1.00e+00	1.00e+06	1.21e+01	5.61e-01	3.75e+00

Figure S6. The binding processes in constant k_{on} and changing k_{off}.

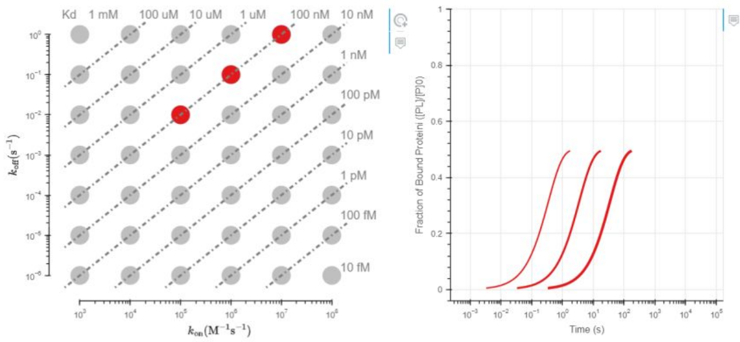

The time to 50% and 99% equilibrium and **[PL]eq** under specific initial conditions (**[P]0** = 100 nM and **[L]0** = 150 nM) and different kinetic parameters in the second-order binding process:

#	Kd (M)	koff (s-1)	kon (M-1 s-1)	[PL]eq (nM)	t0.50 (s)	t0.99 (s)
26	1.00e-07	1.00e-02	1.00e+05	5.00e+01	2.42e+01	1.77e+02
33	1.00e-07	1.00e-01	1.00e+06	5.00e+01	2.42e+00	1.77e+01
40	1.00e-07	1.00e+00	1.00e+07	5.00e+01	2.42e-01	1.77e+00

Figure S7. The binding processes in the same K_d.

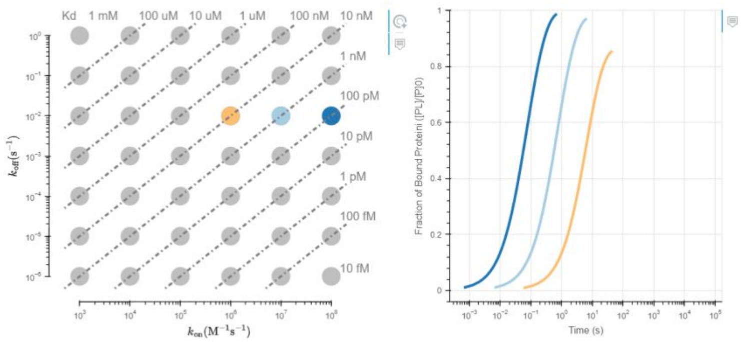

The time to 50% and 99% equilibrium and **[PL]eq** under specific initial conditions (**[P]0** = 100 nM and **[L]0** = 150 nM) and different kinetic parameters in the second-order binding process:

#	Kd (M)	koff (s-1)	kon (M-1 s-1)	[PL]eq (nM)	t0.50 (s)	t0.99 (s)
27	1.00e-08	1.00e-02	1.00e+06	8.64e+01	4.67e+00	4.50e+01
28	1.00e-09	1.00e-02	1.00e+07	9.81e+01	5.59e-01	6.57e+00
29	1.00e-10	1.00e-02	1.00e+08	9.98e+01	5.74e-02	7.00e-01

Figure S8. The binding processes in constant k_{off} and changing k_{on}.

4 Error analysis of the simulation of the competitive binding of association and dissociation

We simulated the competitive binding of association and dissociation with the denominator of the time step being 200,000 and 400,000 respectively in duration of shorter $t_{0.99}$ plus 10×longer $t_{0.99}$ (see Methods). The theoretical $[PL]_{eq\text{-}theo}$ was calculated by Wang equation[1]. In the competitive binding of dissociation, the volume ratio was 1, i.e., the volume of the protein and ligand was the same as the volume of the inhibitor. For example, in exp. No. **1d**, after mixing, the total concentration of the protein, ligand, and inhibitor is 0.1, 1, and 10, respectively. The experiment conditions and results are shown in the Table S1 and S2. In all experiments, the difference between the $[PL]_{eq\text{-}theo}$ and $[PL]_{eq\text{-}sim}$ was less than 0.1% of the $[PL]_{eq\text{-}theo}$.

Table S1. Comparison of the simulated $[PL]_{eq}$ and theoretical $[PL]_{eq}$ under four experiment conditions

Exp. No.	$[P]_0$ (nM)	$[L]_0$ (nM)	$[I]_0$ (nM)	$k_{off\text{-}ligand}$ (s^{-1})	$k_{on\text{-}ligand}$ (M^{-1} s^{-1})	$k_{off\text{-}inhibitor}$ (s^{-1})	$k_{on\text{-}inhibitor}$ (M^{-1} s^{-1})	$[PL]_{eq\text{-}sim}$ (nM)	$[PL]_{eq\text{-}theo}$ (nM)
1a	0.1	1	10	0.01	1e7	0.01	1e6	0.0326360	0.0326370
2a	1	10	100	0.01	1e6	0.1	1e7	0.0833449	0.0832751
3a	10	100	1000	0.1	1e7	0.1	1e6	4.6547598	4.6541926
4a	100	1000	10000	0.1	1e6	1	1e6	46.5319972	46.5419255

Table S2. Comparison of the simulated $[PL]_{eq}$ and theoretical $[PL]_{eq}$ under four experiment conditions

Exp. No.	$[P]_0$ (nM)	$[L]_0$ (nM)	$[I]_0$ (nM)	$k_{off\text{-}ligand}$ (s^{-1})	$k_{on\text{-}ligand}$ (M^{-1} s^{-1})	$k_{off\text{-}inhibitor}$ (s^{-1})	$k_{on\text{-}inhibitor}$ (M^{-1} s^{-1})	$[PL]_{eq\text{-}sim}$ (nM)	$[PL]_{eq\text{-}theo}$ (nM)
1d	0.2	2	20	0.01	1e7	0.01	1e6	0.03263589	0.0326371
2d	2	20	200	0.01	1e6	0.1	1e7	0.0833455	0.0832751
3d	20	200	2000	0.1	1e7	0.1	1e6	4.6541671	4.6541926
4d	200	2000	20000	0.1	1e6	1	1e6	46.5644474	46.5419255

5 Hypothetical association and dissociation kinetic experiments

We first demonstrated two ligands with the same thermodynamic property but different kinetic properties (Table S3). The ligand in experiment No. (hereafter referred to as exp. No.) **1** was fast-on-fast-off, its k_{on} was 1e6 M^{-1} s^{-1} and k_{off} was 0.1 s^{-1}. Compared with exp. No. **1**, the ligand in exp. No. **2** was slow-on-slow-off, its k_{on} was 1e5 M^{-1} s^{-1} and k_{off} was 0.01 s^{-1}.

The K_d of both ligands equaled 100 nM the same as the "K_d in the K_d and apparent K_d can be shown in theoretical saturation curves" section in the article. In exp. No. **1** and **2**, the $[P]_0$ equaled 100 nM, and the $[L]_0$ equaled 150 nM. In both experiments, the equilibrium concentration of the free ligand equaled the K_d, i.e., $[L]_{eq}$ = 100 nM and $[PL]_{eq}$ = 50 nM. The time to reach 99% equilibrium in the second-order binding process ($t_{0.99}$) was 18 seconds and 177 seconds for exp. No. **1** and **2** respectively. If we calculated by using the pseudo-first-order binding process, the time to reach 99% equilibrium ($t_{pseudo\text{-}0.99}$) was 18 seconds and 184 seconds for exp. No. **1** and **2** respectively. The ratio of the $t_{pseudo\text{-}0.99}$ to $t_{0.99}$ ($t_{pseudo\text{-}0.99}/t_{0.99}$=1.06) was 1.04 for both exp. No. **1** and **2**.

In Table S3, as shown by exp. No. **2**, **3**, **4**, and **5**, increasing the $[L]_0$ with the other parameters being constant accelerated the binding process to 99% equilibrium, the $t_{0.99}$ decreased from 177 seconds to 30 seconds. From exp. No. **2** to **5**, both $[PL]_{pseudo\text{-}eq}$ and $[PL]_{eq}$ increased and the $[PL]_{pseudo\text{-}eq}$ approached the $[PL]_{eq}$. When the $[L]_0$ was the largest in exp. No. **5**, the $[PL]_{pseudo\text{-}eq}$ and $[PL]_{eq}$ was the most approximate to each other. This was consistent with the condition "$[L]_0 >> [P]_0$" of the pseudo-first-order binding process. As shown by exp. No. **5**, **6**, **7**, and **8**, increasing either the k_{on} or k_{off} by 10-fold accelerated the binding process to 99% equilibrium, whereas increasing k_{on} had a more obvious effect on the acceleration of the binding process.

For the ligand in exp. No. **1**, the k_{off} was 0.1 s^{-1}, the half-life for dissociation (i.e., $\ln(2)/k_{off}$) equaled 7 seconds and the time to 99% complete dissociation equaled 46 seconds. For the ligand in exp. No. **2**, the k_{off} was 0.01 s^{-1}, the half-life for dissociation equaled 69 seconds and the time to 99% complete dissociation equaled 461 seconds.

Table S3. The association kinetic experiments in different conditions

Exp. No.	$[P]_0$ (nM)	$[L]_0$ (nM)	k_{off} (s^{-1})	k_{on} (M^{-1} s^{-1})	$[PL]_{eq}$ (nM)	$[PL]_{pseudo-eq}$ (nM)	$t_{0.99}$ (s)	$t_{pseudo-0.99}$ (s)	$t_{pseudo-0.99}/t_{0.99}$
**1**	100	150	0.1	1e6	50.0	60.0	17.7	18.4	1.04
**2**	100	150	0.01	1e5	50.0	60.0	177.0	184.2	1.04
**3**	100	300	0.01	1e5	69.7	75.0	122.9	115.1	0.94
**4**	100	600	0.01	1e5	83.8	85.7	70.9	65.8	0.93
**5**	100	1500	0.01	1e5	93.4	93.8	30.0	28.8	0.96
**6**	100	1500	0.1	1e5	59.0	60.0	18.5	18.4	1.00
**7**	100	1500	0.01	1e6	99.3	99.3	3.2	3.0	0.95
**8**	100	1500	0.1	1e6	93.4	93.8	3.0	2.9	0.96

6 Hypothetical competitive binding experiments of association and dissociation

We conducted competitive binding experiments of association and dissociation by simulation (see Table S4). The association and dissociation experiments were specified by #a and #d, using a and d as suffix, respectively. In exp. No. **9a**, the total concentration of the ligand and inhibitor ($[L]_0 + [I]_0$) was the same as the $[L]_0$ in exp. No. **2**. Additionally, the kinetic and thermodynamic properties of the ligand and inhibitor in exp. No. **9a** and the ligand in exp. No. **2** were the same. The time to 99% equilibrium ($t_{0.99}$) of the competitive binding in exp. No. **9a** estimated by numerical simulation was the same as the $t_{0.99}$ of the association process in exp. No. **2** calculated by analytical integration (Table S3 and S4). The consistent $t_{0.99}$ suggested that the competitive binding process could be accurately approximated by numerical simulation. The simulations of four competitive binding experiments also showed that the errors between the simulated $[PL]_{eq}$ and theoretical $[PL]_{eq}$ were less than 0.1% of the theoretical $[PL]_{eq}$ in our default setting (Table S1 and S2).

We changed the concentrations and kinetic parameters in the competitive binding of association to compare the numerical simulation of the second-order process and the analytical integration of the pseudo-first-order process (Table S4). As shown in exp. No. **10a**, we decreased the $[P]_0$ from 100 nM to 10 nM, which was less than the $[L]_0$ and $[I]_0$, with the kinetic properties being constant, the equilibrium concentration ($[PL]_{pseudo\text{-}eq}$) of the protein-ligand complex of the pseudo-first-order binding process approached that of the second-order binding process (the theoretical $[PL]_{eq}$). The $t_{pseudo\text{-}0.99}$ of the pseudo-first-order binding process also approached the $t_{0.99}$ of the second-order binding process. In exp. No. **11a** and **12a**, with the increment of the $[L]_0$ from 75 nM to 150 and 300 nM, and other parameters being constant, the $t_{0.99}$ decreased and the IC_{50} increased. In exp. No. **13a** and **14a**, with the increment of the $[I]_0$ from 75 nM to 150 and 300 nM, and other parameters being constant, the $t_{0.99}$ also decreased. Compared with exp. No. **13a** and **14a**, the $t_{0.99}$ of the slow-on-slow-off inhibitors in exp. No. **15a** and **16a** increased. Furthermore, the larger the $[I]_0$ was, the less the $t_{0.99}$ was. The $t_{0.99}$ of the fast-on-fast-off inhibitors in exp. No. **17a** and **18a** was less than the $t_{0.99}$ in exp. No. **15a** and **16a** (slow-on-

slow-off) but was larger than the $t_{0.99}$ in exp. No. **13a** and **14a** (moderate-on-moderate-off). In contrast to exp. No. **15a** and **16a**, in exp. No. **17a** and **18a**, the larger the $[I]_0$ was, the larger the $t_{0.99}$ was. This suggested that although both ligand and inhibitor had the same thermodynamic property, the different kinetic properties (both fast or slow on and off) could prolong the equilibrium process of the competitive binding. Compared with exp. No. **19a**, if we decreased the K_d (exp. No. **21a**) or K_i (exp. No. **22a**) tenfold or increased the $[L]_0$ tenfold (exp. No. **20a**), the IC_{50} increased similarly tenfold in each of the three experimental changes. From exp. No. **9a** and **9d** to No. **22a** and **22d**, most competitive bindings of dissociation had a larger $t_{0.99}$ than the corresponding competitive bindings of association, except exp. No. **19d**, **20d**, **21d**, and **22d**. The $t_{0.99}$ being 0 meant that the instantaneous concentration of the protein-ligand complex after 1:1 mixing with the inhibitor had already been in the range of [0.99×theoretical $[PL]_{eq}$, 1.01×theoretical $[PL]_{eq}$].

Table S4. Competitive binding experiments of association and dissociation in different conditions[a]

Exp. No.	$[P]_0$ (nM)	$[L]_0$ (nM)	$[I]_0$ (nM)	$k_{off\text{-}ligand}$ (s^{-1})	$k_{on\text{-}ligand}$ (M^{-1} s^{-1})	$k_{off\text{-}inhibitor}$ (s^{-1})	$k_{on\text{-}inhibitor}$ (M^{-1} s^{-1})	$[PL]_{eq}$ (nM)	$[PL]_{pseudo\text{-}eq}$ (nM)	$t_{0.99}$ (s)	$t_{pseudo\text{-}0.99}$ (s)	IC_{50} (nM)	Apparent IC_{50} (nM)
**9a**	100	75	75	0.01	1e5	0.01	1e5	25.00	30.00	1.77e+2	1.84e+2	228.87	287.78
**9d**	100	75	75	0.01	1e5	0.01	1e5	25.00	–	2.90e+2	–	228.87	287.78
**10a**	10	75	75	0.01	1e5	0.01	1e5	2.95	3.00	1.85e+2	1.84e+2	178.86	183.95
**10d**	10	75	75	0.01	1e5	0.01	1e5	2.95	–	4.40e+2	–	178.86	183.95
**11a**	10	150	75	0.01	1e5	0.01	1e5	4.57	4.62	1.43e+2	1.41e+2	251.07	256.11
**11d**	10	150	75	0.01	1e5	0.01	1e5	4.57	–	3.87e+2	–	251.07	256.11
**12a**	10	300	75	0.01	1e5	0.01	1e5	6.29	6.32	9.78e+1	9.61e+1	398.99	404.00
**12d**	10	300	75	0.01	1e5	0.01	1e5	6.29	–	3.23e+2	–	398.99	404.00
**13a**	10	150	150	0.01	1e5	0.01	1e5	3.73	3.75	1.16e+2	1.15e+2	251.07	256.11
**13d**	10	150	150	0.01	1e5	0.01	1e5	3.73	–	4.48e+2	–	251.07	256.11
**14a**	10	150	300	0.01	1e5	0.01	1e5	2.72	2.73	8.44e+1	8.32e+1	251.07	256.11
**14d**	10	150	300	0.01	1e5	0.01	1e5	2.72	–	5.10e+2	–	251.07	256.11
**15a**	10	150	150	0.01	1e5	0.001	1e4	3.73	3.75	2.53e+3	2.63e+3	251.07	256.11
**15d**	10	150	150	0.01	1e5	0.001	1e4	3.73	–	2.63e+3	–	251.07	256.11
**16a**	10	150	300	0.01	1e5	0.001	1e4	2.72	2.73	2.26e+3	2.31e+3	251.07	256.11
**16d**	10	150	300	0.01	1e5	0.001	1e4	2.72	–	2.35e+3	–	251.07	256.11
**17a**	10	150	150	0.01	1e5	0.1	1e6	3.73	3.75	2.84e+2	2.92e+2	251.07	256.11
**17d**	10	150	150	0.01	1e5	0.1	1e6	3.73	–	2.97e+2	–	251.07	256.11
**18a**	10	150	300	0.01	1e5	0.1	1e6	2.72	2.73	3.28e+2	3.36e+2	251.07	256.11
**18d**	10	150	300	0.01	1e5	0.1	1e6	2.72	–	3.85e+2	–	251.07	256.11
**19a**	10	150	75	0.01	1e6	0.1	1e6	8.90	8.96	6.47e+1	6.01e+1	1559.45	1564.46
**19d**	10	150	75	0.01	1e6	0.1	1e6	8.90	–	3.53e+1	–	1559.45	1564.46
**20a**	10	1500	75	0.01	1e6	0.1	1e6	9.88	9.88	1.65e+1	1.65e+1	15044.83	15049.83
**20d**	10	1500	75	0.01	1e6	0.1	1e6	9.88	–	0	–	15044.83	15049.83
**21a**	10	150	75	0.01	1e7	0.1	1e6	9.88	9.88	1.68e+1	1.65e+1	14608.61	14613.61
**21d**	10	150	75	0.01	1e7	0.1	1e6	9.88	–	0	–	14608.61	14613.61
**22a**	10	150	75	0.01	1e6	0.1	1e5	9.29	9.33	3.55e+1	3.42e+1	15604.05	15609.06
**22d**	10	150	75	0.01	1e6	0.1	1e5	9.29	–	1.11e+1	–	15604.05	15609.06
**23a**	30	5	100	0.01	1e6	0.1	1e5	2.95	6.00	1.74e+2	2.39e+2	395.67	418.17
**23d**	30	5	100	0.01	1e6	0.1	1e5	2.95	-	1.50e+2	-	395.67	418.17

[a]For easy comparison, the $[P]_0$, $[L]_0$, and $[I]_0$ in the competitive binding of dissociation have been converted to the total concentration of the protein, ligand, and inhibitor after mixing in the volume ratio of 1.

7 [PL] curves in “Competitive Binding Kinetics – Dissociation” showed S-shape in increasing concentrations of free protein

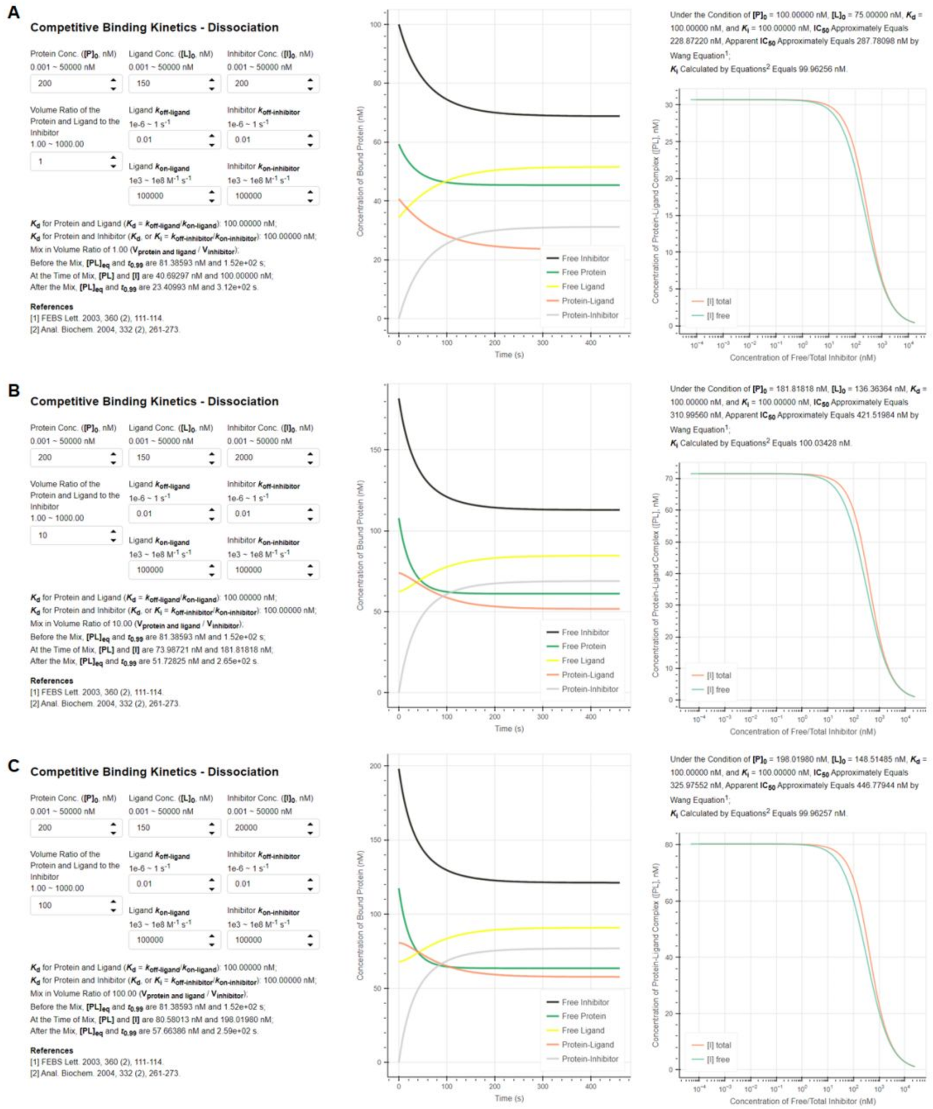

Figure S9. Three screenshots of the customized webpage of Competitive Binding Kinetics – Dissociation. The concentrations of the protein and ligand were 200 and 150 nM, respectively. The k_{off} of the ligand and inhibitor was 0.01 s^{-1}, the k_{on} of the ligand and inhibitor was 1e5 M^{-1} s^{-1}. In each screenshot, the concentration of the inhibitor was 200, 2000, and 20000 nM in (A), (B), and (C). The volume ratio of the protein and ligand to the inhibitor was 1, 10, and 100 in (A), (B), and (C). From (A) to (C), at the time of mixing, the concentration of the free protein increased. the inhibitor could bind more free proteins, so the concentration of the protein-ligand complex decreased more slowly.

8 The calculation of the K_i and apparent K_i

In the competitive binding experiment, the binding of the protein and ligand and the binding of the protein and inhibitor are described by the following equations,

$$\mathrm{P} + \mathrm{L} \overset{K_\mathrm{d}}{\rightleftharpoons} \mathrm{PL}$$

$$\mathrm{P} + \mathrm{I} \overset{K_\mathrm{i}}{\rightleftharpoons} \mathrm{PI}$$

The K_d is the dissociation constant of the protein and ligand. The K_i is the dissociation constant of the protein and inhibitor. At equilibrium, the total concentration of the protein ($[P]_0$) is the sum of the equilibrium concentration of the protein ($[P]_{eq}$), the equilibrium concentration of the protein-ligand complex ($[PL]_{eq}$), and the equilibrium concentration of the protein-inhibitor complex ($[PI]_{eq}$).

$$[\mathrm{P}]_0 = [\mathrm{P}]_\mathrm{eq} + [\mathrm{PL}]_\mathrm{eq} + [\mathrm{PI}]_\mathrm{eq} \quad \textbf{(8.1)}$$

At equilibrium,

$$K_\mathrm{d} = \frac{[\mathrm{P}]_\mathrm{eq}[\mathrm{L}]_\mathrm{eq}}{[\mathrm{PL}]_\mathrm{eq}}$$

$$K_\mathrm{i} = \frac{[\mathrm{P}]_\mathrm{eq}[\mathrm{I}]_\mathrm{eq}}{[\mathrm{PI}]_\mathrm{eq}} \quad \textbf{(8.2)}$$

Equation 8.2 can be written as

$$[\mathrm{PI}]_\mathrm{eq} = \frac{[\mathrm{P}]_\mathrm{eq}[\mathrm{I}]_\mathrm{eq}}{K_\mathrm{i}} \quad \textbf{(8.3)}$$

We substitute equation 8.3 into equation 8.1 and obtain

$$[\mathrm{P}]_0 = \mathrm{P}_\mathrm{eq} + [\mathrm{PL}]_\mathrm{eq} + \frac{[\mathrm{P}]_\mathrm{eq}[\mathrm{I}]_\mathrm{eq}}{K_\mathrm{i}}$$

$$[\mathrm{P}]_0 = [\mathrm{P}]_\mathrm{eq}(1 + \frac{[\mathrm{I}]_\mathrm{eq}}{K_\mathrm{i}}) + [\mathrm{PL}]_\mathrm{eq} \quad \textbf{(8.4)}$$

We multiple $\frac{[\mathrm{L}]_\mathrm{eq}}{[\mathrm{PL}]_\mathrm{eq}}$ on both sides of equation 8.4 and obtain

$$\frac{[\mathrm{P}]_0[\mathrm{L}]_\mathrm{eq}}{[\mathrm{PL}]_\mathrm{eq}} = \frac{[\mathrm{P}]_\mathrm{eq}[\mathrm{L}]_\mathrm{eq}}{[\mathrm{PL}]_\mathrm{eq}}(1 + \frac{[\mathrm{I}]_\mathrm{eq}}{K_\mathrm{i}}) + [\mathrm{L}]_\mathrm{eq}$$

$$\frac{[\mathrm{P}]_0[\mathrm{L}]_\mathrm{eq}}{[\mathrm{PL}]_\mathrm{eq}} = K_\mathrm{d}(1 + \frac{[\mathrm{I}]_\mathrm{eq}}{K_\mathrm{i}}) + [\mathrm{L}]_\mathrm{eq}$$

$$[\mathrm{PL}]_\mathrm{eq} = \frac{[\mathrm{P}]_0[\mathrm{L}]_\mathrm{eq}}{K_\mathrm{d}(1 + \frac{[\mathrm{I}]_\mathrm{eq}}{K_\mathrm{i}}) + [\mathrm{L}]_\mathrm{eq}} \quad \textbf{(8.5)}$$

Equation 8.5 can be transformed into equation 8.6

$$K_\mathrm{i} = \frac{[\mathrm{I}]_\mathrm{eq}}{\frac{[\mathrm{L}]_\mathrm{eq}([\mathrm{P}]_0 - [\mathrm{PL}]_\mathrm{eq})}{[\mathrm{PL}]_\mathrm{eq}K_\mathrm{d}} - 1} \quad \textbf{(8.6)}$$

When 50% of the "initial binding" of the protein and ligand is inhibited, the concentration of the free competing inhibitor ($[\mathrm{I}]_\mathrm{eq}$) is defined as the IC_{50} and the concentration of the total competing inhibitor ($[\mathrm{I}]_0$) is defined as the apparent IC_{50}. The initial binding means the equilibrium concentration of the protein-ligand complex in the blank control in the competitive binding of either association or dissociation. In association, the blank control was the mixture of the protein and the solution with ligand and no inhibitor. In dissociation, the blank control was the mixture of the equilibrated protein and ligand and the solution without inhibitor. The blank control is important to eliminate the equilibrium concentration change of the protein-ligand complex upon the volume change after mixing. At the equilibrium state of 50% inhibition of the protein-ligand binding, the concentration of the protein-ligand complex ($[\mathrm{PI}]_\mathrm{eq\text{-}50}$) can be written as

$$[\mathrm{PL}]_\mathrm{eq\,\text{-}\,50} = \frac{[\mathrm{P}]_0[\mathrm{L}]_\mathrm{eq\,\text{-}\,50}}{K_\mathrm{d}(1 + \frac{[\mathrm{I}]_\mathrm{eq\,\text{-}\,50}}{K_\mathrm{i}}) + [\mathrm{L}]_\mathrm{eq\,\text{-}\,50}} \quad \textbf{(8.7)}$$

In this work, we used the Wang equation[1] to simulate the theoretical inhibition curve, and estimated the IC_{50} and apparent IC_{50} (see Methods), without the restrictions of concentrations.

8.1 Calculation of the apparent K_i by the Cheng-Prusoff equation

At the equilibrium state of 50% inhibition of the protein-ligand binding, if $[\mathrm{L}]_0 \gg [\mathrm{P}]_0$ and $[\mathrm{I}]_0 \gg [\mathrm{P}]_0$, or strictly speaking, $[\mathrm{L}]_0 \gg [\mathrm{PL}]_\mathrm{eq\,\text{-}\,50}$ and $[\mathrm{I}]_0 \gg [\mathrm{PI}]_\mathrm{eq\,\text{-}\,50}$, $[\mathrm{L}]_\mathrm{eq\,\text{-}\,50} = [\mathrm{L}]_0 - [\mathrm{PL}]_\mathrm{eq\,\text{-}\,50} \approx [\mathrm{L}]_0$ and $\mathrm{IC}_{50} = [\mathrm{I}]_\mathrm{eq\,\text{-}\,50} = [\mathrm{I}]_0 - [\mathrm{PI}]_\mathrm{eq\,\text{-}\,50} \approx [\mathrm{I}]_0$, equation 8.7 approximately equals

$$[\mathrm{PL}]_\mathrm{eq\,\text{-}\,50} \approx \frac{[\mathrm{P}]_0[\mathrm{L}]_0}{K_\mathrm{d}(1 + \frac{[\mathrm{I}]_0}{K_\mathrm{i}}) + [\mathrm{L}]_0}$$

Thus, IC_{50} is almost equal to $[\mathrm{I}]_0$. From equation 1.13, we know that in the absence of the inhibitor and when $[\mathrm{L}]_0 \gg [\mathrm{P}]_0$, the equilibrium concentration of the protein-ligand complex ($[\mathrm{PL}]_\mathrm{eq\text{-}0}$) equals

$$[\mathrm{PL}]_\mathrm{eq\,\text{-}\,0} = \frac{[\mathrm{L}]_0[\mathrm{P}]_0}{[\mathrm{L}]_0 + K_\mathrm{d}}$$

At the time of 50% inhibition of the protein-ligand binding, $[\mathrm{PL}]_\mathrm{eq\,\text{-}\,0} = 2 \times [\mathrm{PL}]_\mathrm{eq\,\text{-}\,50}$, so

$$\frac{[\mathrm{L}]_0[\mathrm{P}]_0}{[\mathrm{L}]_0 + K_\mathrm{d}} = \frac{2[\mathrm{P}]_0[\mathrm{L}]_0}{K_\mathrm{d}(1 + \frac{[\mathrm{I}]_0}{K_\mathrm{i}}) + [\mathrm{L}]_0}$$

Thus, we obtain the Cheng-Prusoff equation generalized in the competitive binding (equation 8.8), the $[\mathrm{I}]_0$ in equation 8.8 is the apparent IC_{50}, which approximately equals the IC_{50} in the condition of $[\mathrm{I}]_0 \gg [\mathrm{PI}]_{\mathrm{eq\,\text{-}\,50}}$. The apparent IC_{50} can be estimated by the theoretical inhibition curve. The relationship of the apparent K_i and the $[\mathrm{L}]_0$ under different experimental conditions is shown in Figure 5A in the main text.

$$K_\mathrm{i}^{\mathrm{app1}} = \frac{[\mathrm{I}]_0}{1 + \frac{[\mathrm{L}]_0}{K_\mathrm{d}}} \quad \textbf{(8.8)}$$

8.2 Calculation of the apparent K_i by the Lin-Riggs equation

If we substitute equation 8.5 by $[\mathrm{L}]_{\mathrm{eq}} = [\mathrm{L}]_0 - [\mathrm{PL}]_{\mathrm{eq}}$, we obtain

$$[\mathrm{PL}]_{\mathrm{eq}} = \frac{[\mathrm{P}]_0([\mathrm{L}]_0 - [\mathrm{PL}]_{\mathrm{eq}})}{K_\mathrm{d}(1 + \frac{[\mathrm{I}]_{\mathrm{eq}}}{K_\mathrm{i}}) + ([\mathrm{L}]_0 - [\mathrm{PL}]_{\mathrm{eq}})} \quad \textbf{(8.9)}$$

We multiple $\frac{1}{[\mathrm{L}]_0}$ on both sides of equation 8.9 and obtain

$$\frac{[\mathrm{PL}]_{\mathrm{eq}}}{[\mathrm{L}]_0} = \frac{[\mathrm{P}]_0(1 - \frac{[\mathrm{PL}]_{\mathrm{eq}}}{[\mathrm{L}]_0})}{K_\mathrm{d}(1 + \frac{[\mathrm{I}]_{\mathrm{eq}}}{K_\mathrm{i}}) + [\mathrm{L}]_0(1 - \frac{[\mathrm{PL}]_{\mathrm{eq}}}{[\mathrm{L}]_0})} \quad \textbf{(8.10)}$$

At the equilibrium state of 50% inhibition, equation 8.10 can be written as

$$\frac{[\mathrm{PL}]_{\mathrm{eq\,\text{-}\,50}}}{[\mathrm{L}]_0} = \frac{[\mathrm{P}]_0(1 - \frac{[\mathrm{PL}]_{\mathrm{eq\,\text{-}\,50}}}{[\mathrm{L}]_0})}{K_\mathrm{d}(1 + \frac{[\mathrm{I}]_{\mathrm{eq\,\text{-}\,50}}}{K_\mathrm{i}}) + [\mathrm{L}]_0(1 - \frac{[\mathrm{PL}]_{\mathrm{eq\,\text{-}\,50}}}{[\mathrm{L}]_0})} \quad \textbf{(8.11)}$$

We name the equation (6) in the original paper[2] the "Lin-Riggs equation", which was derived in the context of the competitive inhibition of the protein and labelled nucleic acid. In contrast to $\frac{[\mathrm{PL}]_{\mathrm{eq\,\text{-}\,50}}}{[\mathrm{PL}]_{\mathrm{eq\,\text{-}\,0}}} = 0.5$ in the previous section, $\frac{[\mathrm{PL}]_{\mathrm{eq\,\text{-}\,50}}}{[\mathrm{L}]_0} = 0.5$ in the competitive inhibition of the protein and labelled nucleic acid. Thus, equation 8.11 can be converted to

$$K_\mathrm{i} = \frac{2[\mathrm{I}]_{\mathrm{eq\,\text{-}\,50}}K_\mathrm{d}}{2[\mathrm{P}]_0 - [\mathrm{L}]_0 - 2K_\mathrm{d}} \quad \textbf{(8.12)}$$

The usage condition of the Lin-Riggs equation is $[\mathrm{L}]_0 \approx [\mathrm{PL}]_{\mathrm{eq}}$ and $[\mathrm{I}]_0 \gg [\mathrm{PI}]_{\mathrm{eq}}$. In this condition, $\mathrm{IC}_{50} = [\mathrm{I}]_{\mathrm{eq\,\text{-}\,50}} = [\mathrm{I}]_0 - [\mathrm{PI}]_{\mathrm{eq}-50} \approx [\mathrm{I}]_0$, we can thus use equation 8.13 to calculate the apparent K_i,

$$K_{\mathrm{i}}^{\mathrm{app2}} = \frac{2[\mathrm{I}]_0 K_{\mathrm{d}}}{2[\mathrm{P}]_0 - [\mathrm{L}]_0 - 2K_{\mathrm{d}}} \quad \textbf{(8.13)}$$

The $[I]_0$ in equation 8.13 is the apparent IC_{50}, which approximately equals the IC_{50}. Thus, equation 8.13 is essentially the same as the Lin-Riggs equation. The apparent IC_{50} can be estimated by the theoretical inhibition curve. The relationship of the apparent K_i and the $[L]_0$ under different experimental conditions is shown in Figure 5B in the main text.

It is worth noting that the experimental conditions were different between the calculations using the Cheng-Prusoff equation and the Lin-Riggs equation, because the $[PL]_{eq\text{-}0}$ should be greater than the half of the $[L]_0$ in the calculations using the Lin-Riggs equation. Namely, in the conditions of $[P]_0$ = 50 nM and K_d = 50 nM, $[P]_0$ = 5 nM and K_d = 50 nM, and $[P]_0$ = 5 nM and K_d = 5 nM, the $[PL]_{eq\text{-}0}$ was smaller than $[L]_0/2$. When generating the theoretical inhibition curve with increasing $[L]_0$, the range of the $[L]_0$ was also restricted, because the $[PL]_{eq\text{-}0}$ should be greater than the half of the $[L]_0$.

8.3 Calculation of the K_i by the Wang's group equation

We used the Wang's group equation[3] (equation 8.14) to calculate the K_i. At the equilibrium state of 50% inhibition, $\frac{[\mathrm{PL}]_{\mathrm{eq\,-\,50}}}{[\mathrm{PL}]_{\mathrm{eq\,-\,0}}} = 0.5$. The apparent IC_{50} can be estimated by the theoretical inhibition curve.

$$K_{\mathrm{i}} = \frac{[\mathrm{I}]_{\mathrm{eq\,-\,50}}}{\frac{[\mathrm{L}]_{\mathrm{eq\,-\,50}}}{K_{\mathrm{d}}} + \frac{[\mathrm{P}]_{\mathrm{eq\,-\,0}}}{K_{\mathrm{d}}} + 1} \quad \textbf{(8.14)}$$

For the details of the calculation, see the original paper[3] or the function *calc_ki* in our script *ki_app_calc.py* The relationships of the K_i and the $[L]_0$ under different experimental conditions are shown in Figure 5C and 5D in the main text.

8.4 High-precision calculation of the K_i by the Wang's group equation

We used the Python library mpmath[4] to increase the numerical precision of the functions and variables, see the script *ki_app_calc_mpmath.py* (https://github.com/ydu-sci/Binding_Curve_Viewer/tree/main/scripts) for the source code. In higher numerical precision, the fluctuation of the calculated K_i was not reduced or eliminated, and the fluctuation was the same as that in normal numerical precision. The accuracy of the estimated apparent IC_{50} also affected the calculated

K_i in higher numerical precision (Figure S10).

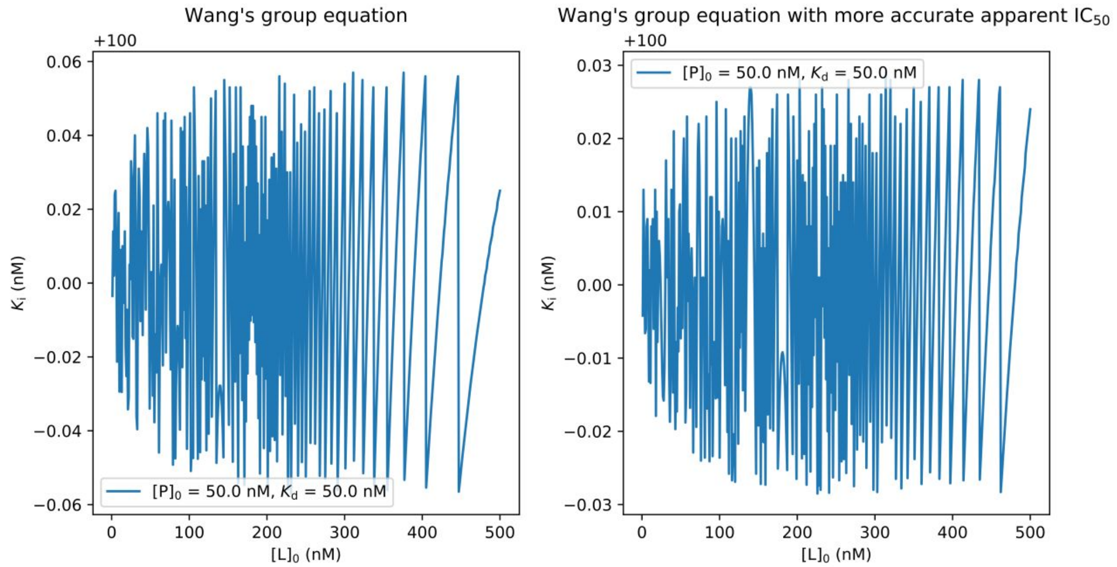

Figure S10. The relationship of the K_i calculated by using the Wang's group equation and the $[L]_0$ in higher precision.

9 Protein and ligand with optimal K_d showed the highest sensitivity in primary screen

To theoretically access the sensitivity of the competitive binding assay in the primary screen, we conducted simulation experiments using different combination of the K_d and K_i. In experimental groups, after mixing, the initial working concentration of the inhibitor was 10 μM. The equilibrium concentration of the protein-ligand complex in blank controls ($[PL]_0$) was 3.0, 5.0 nM, and 7.0 nM, which was respectively 30%, 50%, and 70% of the total concentration of the ligand (10 nM). The K_d was from 1 to 5,000 nM and the K_i was from 1 to 10,000 nM. Both K_d and K_i were increased by a step size of 1, which resulted in a 5000×10000 K_d-K_i matrix.

Under the conditions of $[PL]_0$ = 3.0, 5.0, and 7.0 nM, the ratio of the equilibrium concentrations of the protein-ligand complex in experimental groups ($[PL]_{eq}$) to $[PL]_0$ are shown in Figure S11A-C. With the K_d being constant, inhibitors possessing larger K_i showed lower levels of inhibition (i.e., larger $[PL]_{eq}/[PL]_0$). As the K_d increased, the corresponding K_i on each contour line first increased and then decreased. The increasing and decreasing trends were more obvious in the case of $[PL]_0$ = 7.0 nM. At a preferred level of inhibition, the K_d corresponding to the largest K_i is expected to represent the most sensitive configuration in the primary screen. We graphed the most sensitive K_d at all inhibition levels (Figure S11D-F).

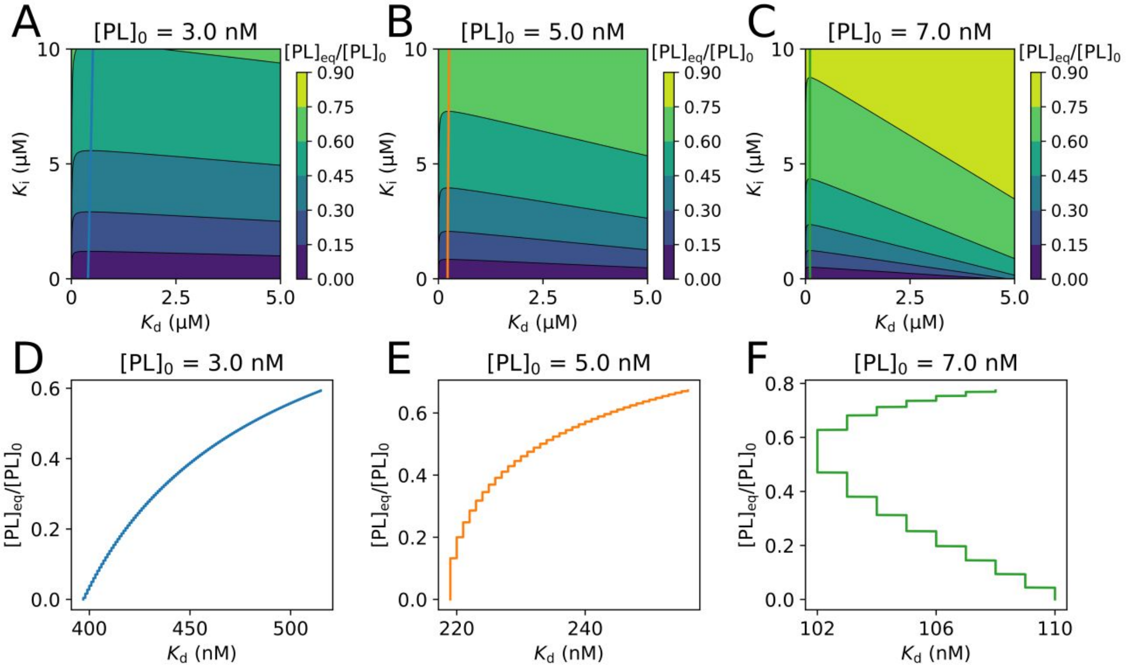

Figure S11. Competitive binding assays in the primary screen under different conditions. The initial working concentration of the inhibitor was 10 μM. (A-C) The contour lines and filled contours of the ratio of the $[PL]_{eq}$ in experimental groups to $[PL]_0$ under the experimental condition of the equilibrium concentration of the protein-ligand complex in blank controls $[PL]_0$ = 3.0, 5.0, and 7.0 nM in the K_d-K_i matrix. The points of the largest K_i on each $[PL]_{eq}/[PL]_0$ contour line were shown in blue, orange, and green line, which are magnified in Figure D, E, and F. (D-F) The relationship of the K_d and the $[PL]_{eq}/[PL]_0$ with the largest K_i in the K_d-K_i matrix under the conditions of $[PL]_0$ = 3.0, 5.0, and 7.0 nM. It should be emphasized that each $[PL]_{eq}/[PL]_0$ value corresponds to a K_d value.

10 Competitive binding experiments under different conditions

We simulated a series of competitive binding experiments under different conditions. We kept the $[PL]_{eq}$ in blank controls 5.0 nM, half of the total concentration of the ligand (10 nM). The relationship between the equilibrium concentration of the protein-ligand complex in experimental groups and the concentration of the inhibitor under different K_d and K_i is shown in Figure S12. At lower K_d values, the apparent IC_{50} was closer to the IC_{50}. When the $[P]_0$ was larger, the more inhibitors were needed to inhibit the binding of the protein and ligand.

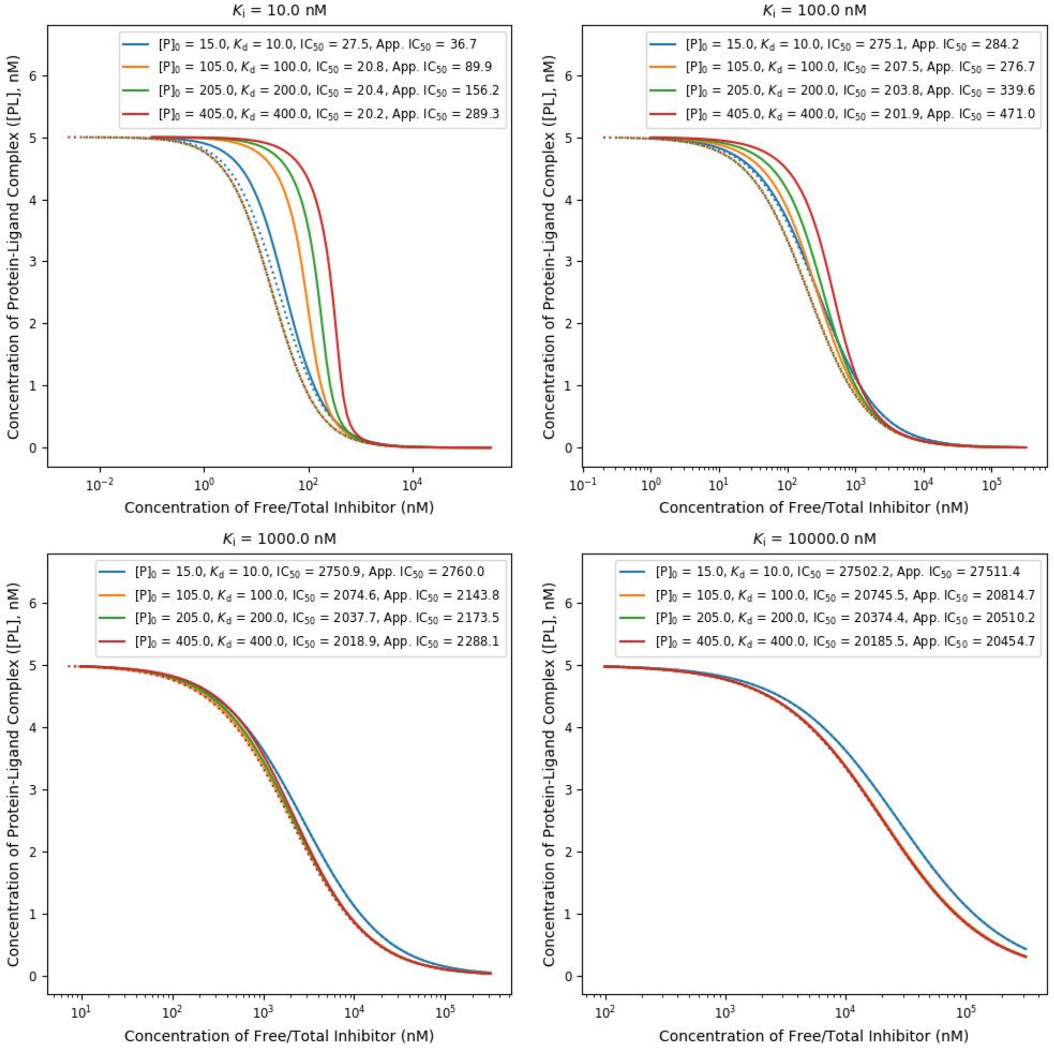

Figure S12. The inhibition curves under different experimental conditions. The total concentration of the ligand was10 nM. In blank controls, the $[PL]_{eq}$ equaled 5.0 nM. The units of all parameters in the legends were nM.

11 Comparison with experimental results

In this section, we reanalyzed some of the data from Jarmoskaite et al.'s radioactive binding assays[5]. In their affinity measurements, the labeled RNA was used as the fixed component, the concentrations of the RNA-binding protein Puf4 were varied. We used the kinetic parameters (in Table 2 of their paper) and related concentrations to calculate the time to reach 99% equilibrium at 25℃ and 0°C (Figure S13 and Table S5). We used the lower limit of the labeled RNA concentration (0.002 nM, see Figure 4 of their paper) as the initial concentration of the fixed component. The gradient concentrations of the Puf4 were 0.001, 0.01, 0.1, 1, 10, and 100 nM. The longest time to reach 99% equilibrium should be used as the incubation time. At 25℃ and 0°C, across all the concentrations of Puf4, the longest time was 3.03e+2 and 1.64e+4 s (i.e., 0.1 and 4.6 h) respectively. Our results of the analysis agree with their results in Section "Time dependence of Puf4 binding at 25°C and 0°C" (see Figure 4 of their paper).

Table S5. The time to reach 99% equilibrium in 2nd-order binding reaction under different experimental conditions.

25℃, k_{off}=0.014 s^{-1}, k_{on}=1e8 M^{-1}s^{-1}, labeled RNA conc.=0.002 nM		0°C, k_{off}=2.92e-5 s^{-1}, k_{on}=2.85e7 M^{-1}s^{-1}, labeled RNA conc.=0.002 nM	
Puf4 conc. (nM)	Time to 99% equilibrium (s)	Puf4 conc. (nM)	Time to 99% equilibrium (s)
**0.01**	3.03e+2	0.01	1.64e+4
**0.1**	1.91e+2	0.1	1.62e+3
**1**	4.04e+1	1	1.62e+2
**10**	4.54e+0	10	1.62e+1
**100**	4.60e-1	100	1.62e+0

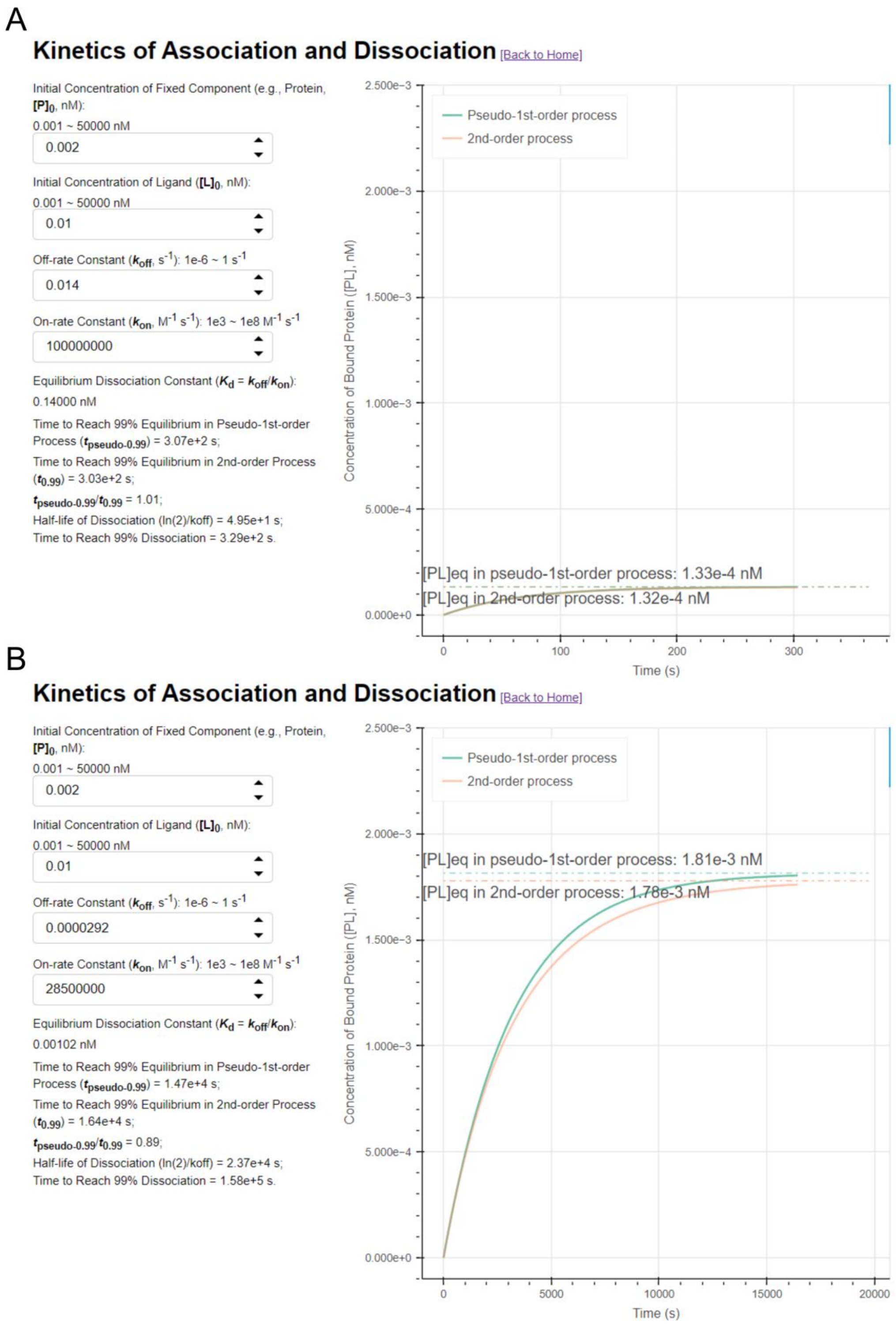

Figure S13. The screenshot of the Binding Curve Viewer – Kinetics of Association and Dissociation. The concentration of the labeled RNA was 0.002 nM. At 25°C (A) and 0°C (B), the time to reach 99% equilibrium in 2nd-order binding reaction was recorded in Table S5 when the gradient concentrations of the Puf4 were 0.001, 0.01, 0.1, 1, 10, and 100 nM.

Next, we analyzed the RNA concentration dependence of Puf4 binding at 25°C and 0°C. We used the lower limit of the labeled RNA concentration shown in Figure 6 of their paper as the initial concentration of the fixed component. The K_d calculated by using the quadratic equation (in Table 2 of their paper) was used in our analysis. At 25°C and 0°C, the K_d was 120 and 1 pM, respectively. Under different labeled RNA concentrations, the apparent K_ds calculated by using Binding Curve Viewer were shown in Table S6. Users can easily compare K_d and apparent K_d with the tool.

Table S6. The apparent K_d calculated by using Binding Curve Viewer under different experimental conditions.

25℃, K_d=120 pM		0℃, K_d=1pM	
RNA conc. (pM)	Apparent K_d (pM)	RNA conc. (pM)	Apparent K_d (pM)
2	121	1	1.5
6	123	3	2.5
18	129	9	5.5
–	–	27	14.5

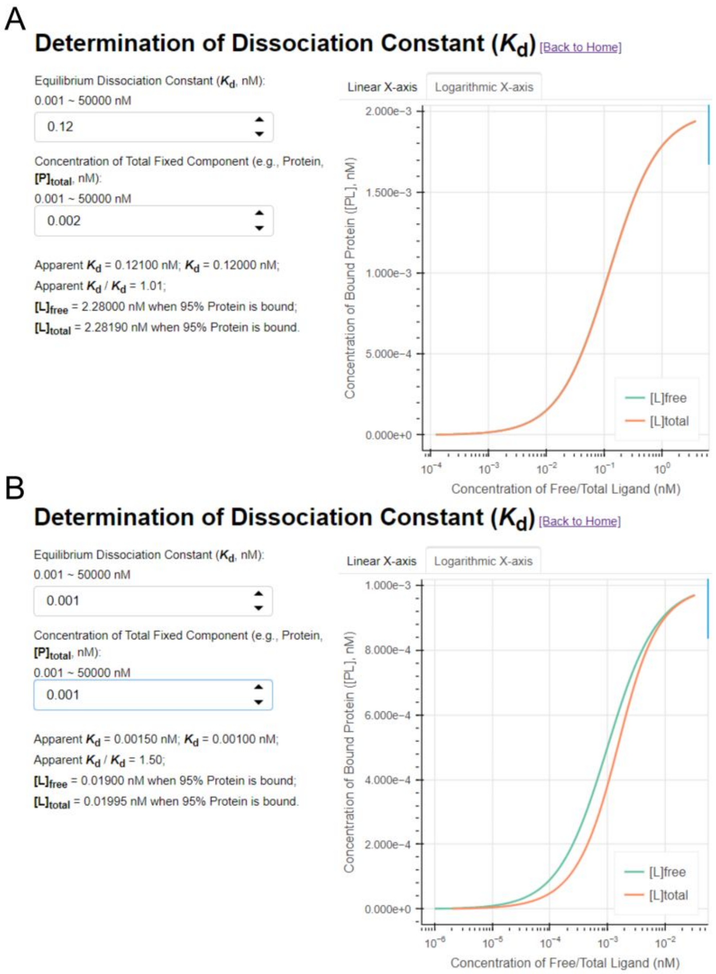

Figure S14. The screenshot of the Binding Curve Viewer – Determination of Dissociation Constant (K_d). At 25°C (A) and 0°C (B), the K_d was 120 and 1 pM, respectively. The labeled RNA was used as the fixed component. The concentrations of the labeled RNA and apparent K_ds were recorded in Table S6.

Reference

1. Wang, Z.-X., An exact mathematical expression for describing competitive binding of two different ligands to a protein molecule. *FEBS Lett.* **1995,** *360* (2), 111-114.
2. Lin, S.-y.; Riggs, A. D., lac represser binding to non-operator DNA: Detailed studies and a comparison of equilibrium and rate competition methods. *J. Mol. Biol.* **1972,** *72* (3), 671-690.
3. Nikolovska-Coleska, Z.; Wang, R.; Fang, X.; Pan, H.; Tomita, Y.; Li, P.; Roller, P. P.; Krajewski, K.; Saito, N. G.; Stuckey, J. A.; Wang, S., Development and optimization of a binding assay for the XIAP BIR3 domain using fluorescence polarization. *Anal. Biochem.* **2004,** *332* (2), 261-273.
4. The mpmath development team *mpmath: a Python library for arbitrary-precision floating-point arithmetic (version 1.2.1)*, 2021.
5. Jarmoskaite, I.; AlSadhan, I.; Vaidyanathan, P. P.; Herschlag, D., How to measure and evaluate binding affinities. *eLife* **2020,** *9*, e57264.
